# Stationary-State Statistics of a Binary Neural Network Model with Quenched Disorder

**DOI:** 10.3390/e21070630

**Published:** 2019-06-26

**Authors:** Diego Fasoli, Stefano Panzeri

**Affiliations:** Laboratory of Neural Computation, Center for Neuroscience and Cognitive Systems@UniTn, Istituto Italiano di Tecnologia, 38068 Rovereto, Italy

**Keywords:** stationary states, multistability, binary neural networks, quenched disorder, bifurcations, extreme value theory, order statistics, matrix permanent, Fisher-Tippett-Gnedenko theorem, Gumbel distribution

## Abstract

In this paper, we study the statistical properties of the stationary firing-rate states of a neural network model with quenched disorder. The model has arbitrary size, discrete-time evolution equations and binary firing rates, while the topology and the strength of the synaptic connections are randomly generated from known, generally arbitrary, probability distributions. We derived semi-analytical expressions of the occurrence probability of the stationary states and the mean multistability diagram of the model, in terms of the distribution of the synaptic connections and of the external stimuli to the network. Our calculations rely on the probability distribution of the bifurcation points of the stationary states with respect to the external stimuli, calculated in terms of the permanent of special matrices using extreme value theory. While our semi-analytical expressions are exact for any size of the network and for any distribution of the synaptic connections, we focus our study on networks made of several populations, that we term “statistically homogeneous” to indicate that the probability distribution of their connections depends only on the pre- and post-synaptic population indexes, and not on the individual synaptic pair indexes. In this specific case, we calculated analytically the permanent, obtaining a compact formula that outperforms of several orders of magnitude the Balasubramanian-Bax-Franklin-Glynn algorithm. To conclude, by applying the Fisher-Tippett-Gnedenko theorem, we derived asymptotic expressions of the stationary-state statistics of multi-population networks in the large-network-size limit, in terms of the Gumbel (double exponential) distribution. We also provide a Python implementation of our formulas and some examples of the results generated by the code.

## 1. Introduction

Biological networks are typically endowed with synaptic connections that have highly heterogeneous strength [1]. The magnitude of the synaptic strength is determined by several variable factors. In the case of chemical synapses, these factors include the size of the available vesicle pool, the release probability of neurotransmitters into the synaptic cleft, the amount of neurotransmitter that can be absorbed in the postsynaptic neuron, and the efficiency of neurotransmitter replenishment [1,2,3,4,5]. These factors differ from synapse to synapse, causing heterogeneity in the distribution of synaptic strengths. An additional source of heterogeneity in biological networks is represented by the number of connections made by the axon terminals to the dendrites of post-synaptic neurons, which affects both the magnitude of the synaptic strengths and the local topology of neural circuits [5]. Heterogeneity of synapses is thought to be an intrinsic network property that cannot be explained by differences across individuals or experiments [4]. It has also been hypothesized that this heterogeneity has an important functional role in making circuits more robust against possible dysfunctions [6].

Synaptic heterogeneity can be included in network models by assuming that the strengths of synapses within the network are random variables with some specific distribution, for example a Gaussian distribution. The standard deviation of the distribution can be interpreted as a measure of how much synapses are heterogeneous.

A powerful way to study the effect of heterogeneities of the interactions within a physical system is *quenched disorder*. In condensed matter physics, quenched disorder describes mathematically the highly irregular atomic bond structure of amorphous solids known as *spin glasses* [7,8,9,10,11,12]. Models with quenched disorder allow the computation of statistically representative and thus physically relevant results by averaging physical observables over the variability of the connections between the network units [12]. This is done by first generating several copies or repetitions of the network, where the strength and/or the topology of the connections are generated randomly from given probability distributions. Each copy owns a different set of frozen connections, which affect the value of physical observables. The physical observables therefore present a probability distribution over the set of connections. Since measurements of macroscopic observables are dominated by their mean values [12], the final, and often difficult step is to calculate averages over the distribution of the connections.

So far, only a few studies have investigated the dynamics of neural networks with heterogeneous, randomly distributed synaptic connections. In [13,14,15], the authors discussed the statistical and dynamical consequences of excitation-inhibition balance, clustering and spatially dependent recurrent connections in networks of integrate-and-fire neurons with random sparse connectivity. Analytical results for graded-rate neurons have been derived in [16,17,18,19,20], with special focus on the thermodynamic limit of fully connected networks. The neurons in these models are all-to-all connected with unit probability, namely the network topology is deterministic, while the strength of the synaptic connections is normally distributed. Other papers have focused on networks of binary neurons in the thermodynamic limit, and taken directly advantage of the powerful statistical approaches developed for studying spin glasses. These approaches can be adapted for the investigation of networks of binary-rate neurons in the thermodynamic limit, to study neural networks endowed with Hebbian plasticity rules for their synaptic connections, that behave as attractor neural networks storing and retrieving desired patterns of neural activity (see [21] and references therein).

Here we aim to extend the study of binary network models with quenched disorder to the case of networks of arbitrary size, paying special attention to small sizes. Studying networks of arbitrary size, including small ones, is important because sizes of brains, and of specialized neural networks within brains, change considerably across animal species, ranging from a few tens of neurons in invertebrates such as rotifers and nematodes, to billions of neurons in cetaceans and primates [22]. Network size also changes across levels of spatial organization, ranging from microscopic and mesoscopic levels of organization in cortical micro-columns and columns (including from a few tens [23] to a few tens of thousands of neurons [24,25]), to several orders of magnitude more in large macroscopic networks [26,27]. The study of how finite-size systems behave with quenched disorder is particularly challenging because each network realization may be very different from another when considering systems of small size. As a result, little is known about the effect of synaptic heterogeneities for finite-size networks.

To perform this study, we build on our recent progress, [28], in which we introduced optimized algorithms for investigating changes in the long-time dynamics of arbitrary-size recurrent networks, composed of binary-rate neurons that evolve in discrete time steps. These changes of dynamics are known in mathematical terms as *bifurcations* [29]. In particular, in [28] we studied changes in the number of stationary network states and the formation of neural oscillations, elicited by variations in the external stimuli to the network. The synaptic strengths and the topology of the network were arbitrary (possibly random and asymmetric), and we studied the bifurcation structure of single network realizations without averaging it over several copies of the model.

In the present paper, we extend the mathematical formalism and the algorithms introduced in [28], by deriving a complete semi-analytical description of the statistical properties of the long-time network states and of the corresponding bifurcations, across network realizations. For simplicity, we focus on the mathematical characterization of the stationary states of random networks. Going beyond classic spin-glass theory [7,8,9,10,11,12], we extend our work beyond the calculation of the expected values, by deriving complete probability distributions. Moreover, unlike previous work on random networks of graded neurons [16,17,18,19,20], which focused on fully connected models with normally distributed synaptic strengths in the thermodynamic limit, we investigate the more complicated case of arbitrary-size networks, with arbitrary distribution of the synaptic strengths and of the topology of the connections. Moreover, rather than considering the problem of storing/retrieving some desired sequences of neural activity patterns [21], we determine the fixed-point attractors, namely the stationary solutions of the network equations, that are generated by some given arbitrary distribution of the synaptic connections (i.e., by connections that are not necessarily designed to store and retrieve some desired patterns).

## 2. Materials and Methods

### 2.1. The Network Model

We study a recurrent neural network model composed of *N* binary neurons, whose state evolves in discrete time steps. The firing rate of the *i*th neuron at time *t* is represented by the binary variable $\nu_{i}\left(t\right)\in \left\{0,1\right\}$, so that $\nu_{i}\left(t\right)=0$ if the neuron is not firing at time *t*, and $\nu_{i}\left(t\right)=1$ if it is firing at the maximum rate, see e.g., [30]. We also define $\nu \left(t\right)\overset{\mathrm{def}}{=}{\left[\nu_{0}\left(t\right),\;\nu_{1}\left(t\right),\;\cdots ,\nu_{N-1}\left(t\right)\right]}^{T}\in {\left\{0,1\right\}}^{N}$, namely the vector containing the firing rates of all *N* neurons at time *t*.

If the neurons respond synchronously to the local fields $h_{i}\left(\nu \left(t\right)\right)$, the firing rates at the time instant t+1 are updated according to the following activity-based equation (see e.g., [28,31]):

(1) $$\nu_{i}\left(t+1\right)=H\left(h_{i}\left(\nu \left(t\right)\right)-\theta_{i}\right),\;h_{i}\left(\nu \left(t\right)\right)\overset{\mathrm{def}}{=}\sum_{j=0}^{N-1}J_{i,j}\nu_{j}\left(t\right)+I_{i},\;i=0,\cdots ,N-1.$$

As we anticipated, *N* is the number of neurons in the network, which in this work is supposed to be finite. Moreover, $I_{i}$ is a deterministic external input (i.e., an afferent stimulus) to the *i*th neuron, while $H\left(\cdot \right)$ is the Heaviside step function:

$$H\left(h-\theta \right)=\left\{\begin{array}{cc} 0, & \mathrm{if}\;\;h<\theta \\ 1, & \mathrm{if}\;\;h\geq \theta , \end{array}\right.$$

with deterministic firing threshold θ.

$J_{i,j}$ is the (generally asymmetric) entry of the synaptic connectivity matrix *J*, and represents the strength or weight of the random and time-independent synaptic connection from the *j*th (presynaptic) neuron to the *i*th (postsynaptic) neuron. The randomness of the synaptic weights is *quenched*, therefore a “frozen” connectivity matrix *J* is randomly generated at every realization of the network, according to the following formula:

(2) $$J_{i,j}=\left\{\begin{array}{cc} 0, & \mathrm{if}\;\;T_{i,j}=0\\ W_{i,j}, & \mathrm{if}\;\;T_{i,j}=1. \end{array}\right.$$

In Equation (2), $T_{i,j}$ is the $\left(i,j\right)$th entry of the topology matrix *T*, so that $T_{i,j}=0$ if there is no synaptic connection from the *j*th neuron to the *i*th neuron, and $T_{i,j}=1$ if the connection is present. The topology of the network is generally random and asymmetric, and it depends on the (arbitrary) entries $P_{i,j}\in \left[0,1\right]$ of the connection probability matrix P. In particular, we suppose that $T_{i,j}=1$ with probability $P_{i,j}$, while $T_{i,j}=0$ with probability $1-P_{i,j}$. Moreover, in Equation (2) the terms $W_{i,j}$ are also (generally asymmetric and non-identically distributed) random variables, distributed according to marginal probability distributions $p_{W_{i,j}}$ (for simplicity, here we focus on continuous distributions, however our calculations can be extended to discrete random variables, if desired). To ensure the mathematical tractability of the model, we suppose that the terms $W_{i,j}$ are statistically independent from each other and from the variables $T_{i,j}$, and that the variables $T_{i,j}$ are independent too.

On the other hand, if the neurons respond asynchronously to the local fields, at each time instant only a single, randomly drawn neuron *k* is to undergo an update (see [28,31]):

(3) $$\left\{\begin{array}{cc} \nu_{i}\left(t+1\right)=\nu_{i}\left(t\right), & \forall i\neq k\\ \nu_{i}\left(t+1\right)=H\left(h_{i}\left(\nu \left(t\right)\right)-\theta_{i}\right), & i=k, \end{array}\right.$$

where the local field $h_{i}\left(\nu \left(t\right)\right)$ is defined as in Equation (1) (this is known as *Glauber dynamics* [32] in the zero-noise limit). The results that we derive in this paper are valid for both kinds of network updates, synchronous and asynchronous, since they generate identical bifurcation diagrams of the stationary states for a given set of network parameters, as we proved in [28].

For simplicity, from now on we will represent the vector $\nu \left(t\right)$ by the binary string $\nu_{0}\left(t\right)\nu_{1}\left(t\right)\cdots \nu_{N-1}\left(t\right)$, obtained by concatenating the firing rates at time *t*. For example, in a network composed of N=6 neurons, the vector $\nu \left(t\right)={\left[1,\;1,\;0,\;0,\;1,\;0\right]}^{T}$ will be represented by the string 110010 (in this notation, no multiplication is intended between the bits).

### 2.2. Statistical Properties of the Network Model

In this paper, we focus on the calculation of the statistical properties of the stationary solutions of Equations (1) and (3), provided the probability distribution of the entries $J_{i,j}$ of the connectivity matrix is known. Therefore we do not consider the problem of storing/retrieving some desired sequences of neural activity patterns.

Our formalism is based on a branch of statistics known as *extreme value theory* [33], which deals with the extreme deviations from the median of probability distributions. Formally, extreme deviations are described by the minimum and maximum of a set of random variables, which correspond, respectively, to the smallest and largest *order statistics* of that set [34,35,36,37].

In this section, we derive semi-analytical formulas of the probability distribution of the bifurcation points of the stationary states (Section 2.2.1), of their mean multistability diagram (Section 2.2.2), of the probability that a state is stationary for a given combination of stimuli (Section 2.2.3), and of the probability that a state is stationary regardless of the stimuli (Section 2.2.4). We implemented these formulas in the “Semi-Analytical Calculations” section of the Supplemental Python script “Statistics.py”. Note that our formulas are semi-analytical, in that they are expressed in terms of 1D definite integrals containing the arbitrary probability distributions $p_{W_{i,j}}$. In the Python script, these integrals are calculated through numerical integration schemes. However, note that generally the integrals may be calculated through analytical approximations, while for some distributions exact formulas may also exist, providing a fully analytical description of the statistical properties of the stationary states.

#### 2.2.1. Probability Distribution of the Bifurcation Points

The *multistability diagram* of the network provides a complete picture of the relationship between the stationary solutions of the network and a set of network parameters. In particular, in [28] we studied how the degree of multistability (namely the number of stationary solutions) of the firing-rate states and their symmetry depend on the stimuli $I_{i}$, which represent our bifurcation parameters. Given a network with P distinct input currents (i.e., $I_{i}\in \left\{I_{0},\cdots ,I_{P-1}\right\}\;\forall i$), we define $\Gamma_{I_{\alpha }}$ to be the set of neurons that share the same external current $I_{\alpha }$ (namely $\Gamma_{I_{\alpha }}\overset{\mathrm{def}}{=}\left\{i\in \left\{0,\cdots ,N-1\right\}:\;I_{i}=I_{\alpha }\right\}$), while $\Gamma_{I_{\alpha },u}^{\left(\nu \right)}\overset{\mathrm{def}}{=}\left\{i\in \Gamma_{I_{\alpha }}:\;\nu_{i}=u\right\}$, for $u\in \left\{0,1\right\}$. Then in [28] we proved that a given firing-rate state ν is a stationary solution of the network Equations (1) and (3) for every combination of stimuli $I\overset{\mathrm{def}}{=}\left(I_{0},\cdots ,I_{P-1}\right)\in V^{\left(\nu \right)}=V_{0}^{\left(\nu \right)}\times \cdots \times V_{P-1}^{\left(\nu \right)}$, where:

(4) $$\begin{array}{c} V_{\alpha }^{\left(\nu \right)}\overset{\mathrm{def}}{=}\left\{\begin{array}{cc} \left(-\infty ,\Xi_{\alpha }^{\left(\nu \right)}\right), & \mathrm{if}\;\;\Gamma_{I_{\alpha },1}^{\left(\nu \right)}=\emptyset \\ \left[\Lambda_{\alpha }^{\left(\nu \right)},+\infty \right), & \mathrm{if}\;\;\Gamma_{I_{\alpha },0}^{\left(\nu \right)}=\emptyset \\ \left[\Lambda_{\alpha }^{\left(\nu \right)},\Xi_{\alpha }^{\left(\nu \right)}\right), & \mathrm{otherwise} \end{array}\right.\\ \Lambda_{\alpha }^{\left(\nu \right)}\overset{\mathrm{def}}{=}\max_{i\in \Gamma_{I_{\alpha },1}^{\left(\nu \right)}}I_{i}^{\left(\nu \right)},\;\Xi_{\alpha }^{\left(\nu \right)}\overset{\mathrm{def}}{=}\min_{i\in \Gamma_{I_{\alpha },0}^{\left(\nu \right)}}I_{i}^{\left(\nu \right)}\\ I_{i}^{\left(\nu \right)}\overset{\mathrm{def}}{=}\theta_{i}-\sum_{j=0}^{N-1}J_{i,j}\nu_{j}. \end{array}$$

By calculating the hyperrectangles $V^{\left(\nu \right)}$ for every ν, we obtain a complete picture of the relationship between the stationary states and the set of stimuli. If the hyperrectangles corresponding to M different states ν overlap, the overlapping region has multistability degree M (i.e., for combinations of stimuli lying in that region, the network has M distinct stationary states). A stationary state loses its stability at the boundaries $\Lambda_{\alpha }^{\left(\nu \right)}$ and $\Xi_{\alpha }^{\left(\nu \right)}$, turning into another stationary state or an oscillation. Therefore $\Lambda_{\alpha }^{\left(\nu \right)}$ and $\Xi_{\alpha }^{\left(\nu \right)}$ represent the *bifurcation points* of the stationary solution ν of Equations (1) and (3).

In what follows, we derive the probability density functions of the bifurcation points *given* the firing-rate state ν (note that, for simplicity, from now on we will omit the superscript $\left(\nu \right)$ in all the formulas). We define the permanent of an n×n matrix *A* as follows [38]:

$$\mathrm{per}\left(A\right)=\sum_{\sigma }\prod_{i=0}^{n-1}A_{i,\sigma \left(i\right)},$$

where the summation is over the n! permutations σ of the set $\left\{0,\;1,\;\cdots ,\;n-1\right\}$, while $\sigma \left(i\right)$ represents the *i*th position of this set after the reordering (e.g., for n=4 and $\sigma =\left[1,\;3,\;2,\;0\right]$, we get $\sigma \left(1\right)=3$). Since the random variables $I_{i}$ are conditionally independent given the firing-rate state ν (as a consequence of the independence of the synaptic weights $J_{i,j}$), if we call $F_{I_{i}}\left(x\right)\overset{\mathrm{def}}{=}\int_{-\infty }^{x}p_{I_{i}}\left(y\right)dy$ the cumulative distribution function of $I_{i}$, then from Equation (4) we obtain that $\Lambda_{\alpha }$ and $\Xi_{\alpha }$ given ν are distributed according to the following order statistics [34,35,36,37]:

(5) $$\begin{array}{cc} p_{\Lambda_{\alpha }}\left(x\right)= & \frac{1}{\left(\gamma_{\alpha ,1}-1\right)!}\mathrm{per}\left(\left[p_{\alpha ,1}\left(x\right),\;F_{\alpha ,1}^{\left(\gamma_{\alpha ,1}-1\right)}\left(x\right)\right]\right)\\ p_{\Xi_{\alpha }}\left(x\right)= & \frac{1}{\left(\gamma_{\alpha ,0}-1\right)!}\mathrm{per}\left(\left[p_{\alpha ,0}\left(x\right),\;I_{\gamma_{\alpha ,0},\gamma_{\alpha ,0}-1}-F_{\alpha ,0}^{\left(\gamma_{\alpha ,0}-1\right)}\left(x\right)\right]\right), \end{array}$$

where $\gamma_{\alpha ,u}\overset{\mathrm{def}}{=}\left|\Gamma_{I_{\alpha },u}\right|$, while $\left[p_{\alpha ,1}\left(x\right),\;F_{\alpha ,1}^{\left(\gamma_{\alpha ,1}-1\right)}\left(x\right)\right]$ and $\left[p_{\alpha ,0}\left(x\right),\;I_{\gamma_{\alpha ,0},\gamma_{\alpha ,0}-1}-F_{\alpha ,0}^{\left(\gamma_{\alpha ,0}-1\right)}\left(x\right)\right]$ are $\gamma_{\alpha ,1}\times \gamma_{\alpha ,1}$ and $\gamma_{\alpha ,0}\times \gamma_{\alpha ,0}$ matrices respectively, $p_{\alpha ,u}\left(x\right)\overset{\mathrm{def}}{=}{\left[p_{I_{i}}\left(x\right)\right]}_{i\in \Gamma_{I_{\alpha },u}}$ and $F_{\alpha ,u}\left(x\right)\overset{\mathrm{def}}{=}{\left[F_{I_{i}}\left(x\right)\right]}_{i\in \Gamma_{I_{\alpha },u}}$ are $\gamma_{\alpha ,u}\times 1$ column vectors, $F_{\alpha ,u}^{\left(v\right)}\left(x\right)\overset{\mathrm{def}}{=}\underset{v-\mathrm{times}}{\left[\underset{︸}{F_{\alpha ,u}\left(x\right),\;\cdots ,\;F_{\alpha ,u}\left(x\right)}\right]}$ is a $\gamma_{\alpha ,u}\times v$ matrix, and $I_{\gamma_{\alpha ,0},\gamma_{\alpha ,0}-1}$ is the $\gamma_{\alpha ,0}\times \left(\gamma_{\alpha ,0}-1\right)$ all-ones matrix. Please note that in the Supplemental Python script “Statistics.py” the permanent is calculated by means of the Balasubramanian-Bax-Franklin-Glynn (BBFG) formula, which is the fastest known algorithm for the numerical calculation of the permanent of arbitrary matrices [39,40,41,42].

According to Equation (5), in order to complete the derivation of the probability densities $p_{\Lambda_{\alpha }}$ and $p_{\Xi_{\alpha }}$, we need to evaluate the probability density and the cumulative distribution function of $I_{i}$. By defining $S_{i}\overset{\mathrm{def}}{=}\sum_{j=0}^{N-1}J_{i,j}\nu_{j}=\sum_{j\in R}J_{i,j}$ and $R\overset{\mathrm{def}}{=}\left\{j\in \left\{0,\cdots ,N-1\right\}:\;\nu_{j}=1\right\}$, from the definition of $I_{i}$ in Equation (4) it follows that:

(6) $$p_{I_{i}}\left(x\right)=p_{S_{i}}\left(\theta_{i}-x\right).$$

Since the synaptic weights $J_{i,j}$ are independent by hypothesis, the probability distribution of $S_{i}$ can be calculated through the convolution formula:

(7) $$p_{S_{i}}\left(\theta_{i}-x\right)=\left(\underset{j\in R}{*}p_{J_{i,j}}\right)\left(\theta_{i}-x\right),$$

According to Equation (2):

(8) $$p_{J_{i,j}}\left(x\right)=P_{i,j}p_{W_{i,j}}\left(x\right)+\left(1-P_{i,j}\right)\delta \left(x\right),$$

where $\delta \left(\cdot \right)$ is the Dirac delta function. Then, from Equations (7) and (8), it follows that:

(9) $$\begin{array}{cc} p_{S_{i}}\left(\theta_{i}-x\right)= & a_{i}\left(\theta_{i}-x\right)+b_{i}\delta \left(\theta_{i}-x\right)\\ a_{i}\left(x\right)= & \sum_{S\in P\left(R\right)\backslash \emptyset }\left[\prod_{j\in S}P_{i,j}\right]\left[\prod_{j\in R\backslash S}\left(1-P_{i,j}\right)\right]\left[\left(\underset{j\in S}{*}p_{W_{i,j}}\right)\left(x\right)\right]\\ b_{i}= & \prod_{j\in R}\left(1-P_{i,j}\right), \end{array}$$

where $P\left(R\right)$ represents the power set of *R*. Finally, we obtain $p_{I_{i}}\left(x\right)$ from Equations (6) and (9), while the corresponding cumulative distribution function is:

(10) $$F_{I_{i}}\left(x\right)=\int_{-\infty }^{x}a_{i}\left(\theta_{i}-y\right)dy+b_{i}H\left(x-\theta_{i}\right).$$

Please note that the definite integral in Equation (10) depends on the probability distribution $p_{W_{i,j}}$, which is arbitrary. For this reason, we do not provide any analytical expression of this integral, though exact formulas exist for specific distributions $p_{W_{i,j}}$, e.g., when $W_{i,j}$ are normally distributed (in the Supplemental Python script “Statistics.py”, the distribution $p_{W_{i,j}}$ is defined by the user, and the integrals are calculated by means of numerical integration schemes).

Now we have all the ingredients for calculating the probability distributions of the bifurcation points from Equation (5). Note, however, that this formula cannot be used in its current form, because it involves the ill-defined product between the Dirac delta function (which is contained in the probability density $p_{I_{i}}$) and a discontinuous function (i.e., $F_{I_{i}}$, which jumps at $x=\theta_{i}$). To see that this product is generally ill-defined, consider for example the probability density of $\min_{j}\left(X_{j}\right)$, in the case when $X_{j}$ are *n* independent and identically distributed random variables:

(11) $$p_{\min_{j}\left(X_{j}\right)}\left(x\right)=\frac{1}{\left(n-1\right)!}\mathrm{per}\left(\left[\begin{array}{c} p_{X}\left(x\right)\\ \vdots \\ p_{X}\left(x\right) \end{array}\underset{\left(n-1\right)-\mathrm{times}}{\underset{︸}{\begin{array}{ccc} F_{X}\left(x\right) & \cdots & F_{X}\left(x\right)\\ \vdots & \ddots & \vdots \\ F_{X}\left(x\right) & \cdots & F_{X}\left(x\right) \end{array}}}\right]\right)=np_{X}\left(x\right)F_{X}^{n-1}\left(x\right).$$

If $F_{X}\left(x\right)$ is absolutely continuous, we can prove that $p_{\min_{j}\left(X_{j}\right)}\left(x\right)$, as given by Equation (11), is correctly normalized:

$$\int_{-\infty }^{+\infty }np_{X}\left(x\right)F_{X}^{n-1}\left(x\right)dx=\int_{-\infty }^{+\infty }n\frac{dF_{X}\left(x\right)}{dx}F_{X}^{n-1}\left(x\right)dx=\int_{0}^{1}nF_{X}^{n-1}dF_{X}={\left.n\frac{1}{n}F_{X}^{n}\right|}_{0}^{1}=1.$$

However, in the limit $p_{X}\left(x\right)\to \delta \left(x\right)$, the cumulative distribution function $F_{X}\left(x\right)$ is discontinuous at x=0, and we get:

$$\int_{-\infty }^{+\infty }np_{X}\left(x\right)F_{X}^{n-1}\left(x\right)dx\to \int_{-\infty }^{+\infty }n\delta \left(x\right)H^{n-1}\left(x\right)dx.$$

If now we attempt to apply the famous formula:

$$\int_{-\infty }^{+\infty }\delta \left(x\right)f\left(x\right)dx=f\left(0\right),$$

we get that the integral equals $nH^{n-1}\left(0\right)=n>1$, therefore $p_{\min_{j}\left(X_{j}\right)}\left(x\right)$ is not properly normalized. The same problem occurs for $\max_{j}\left(X_{j}\right)$.

To fix it, consider the general case when *X* is a mixture of continuous and discrete random variables. Its probability density can be decomposed as follows:

(12) $$p_{X}\left(x\right)=p_{X^{c}}\left(x\right)+\sum_{q\in D}\left[F_{X}\left(x_{q}\right)-\lim_{x\to x_{q}^{-}}F_{X}\left(x\right)\right]\delta \left(x-x_{q}\right).$$

In Equation (12), $p_{X^{c}}$ is the component of $p_{X}$ that describes the statistical behavior of the continuous values of *X*. Moreover, ${\left\{x_{q}\right\}}_{q\in D}$ represents the set of the discrete values of *X*, at which the cumulative distribution function $F_{X}$ is (possibly) discontinuous. In the specific case when $X=\Lambda_{\alpha }$ or $X=\Xi_{\alpha }$, by comparing Equation (12) with Equations (5), (6) and (9), we get:

(13) $$\begin{array}{cc} p_{\Lambda_{\alpha }^{c}}\left(x\right)= & \frac{1}{\left(\gamma_{\alpha ,1}-1\right)!}\mathrm{per}\left(\left[a_{\alpha ,1}\left(\theta -x\right),\;F_{\alpha ,1}^{\left(\gamma_{\alpha ,1}-1\right)}\left(x\right)\right]\right)\\ p_{\Xi_{\alpha }^{c}}\left(x\right)= & \frac{1}{\left(\gamma_{\alpha ,0}-1\right)!}\mathrm{per}\left(\left[a_{\alpha ,0}\left(\theta -x\right),\;I_{\gamma_{\alpha ,0},\gamma_{\alpha ,0}-1}-F_{\alpha ,0}^{\left(\gamma_{\alpha ,0}-1\right)}\left(x\right)\right]\right), \end{array}$$

where $a_{\alpha ,u}\left(\theta -x\right)\overset{\mathrm{def}}{=}{\left[a_{i}\left(\theta_{i}-x\right)\right]}_{i\in \Gamma_{I_{\alpha },u}}$ is a $\gamma_{\alpha ,u}\times 1$ column vector. Moreover, $D=\Gamma_{I_{\alpha },1}$ and $D=\Gamma_{I_{\alpha },0}$ for $\Lambda_{\alpha }$ and $\Xi_{\alpha }$ respectively, while $x_{q}=\theta_{q}$. According to Equation (12), we also need to evaluate the cumulative distribution functions of $\Lambda_{\alpha }$ and $\Xi_{\alpha }$. By following [36], we get:

(14) $$\begin{array}{cc} F_{\Lambda_{\alpha }}\left(x\right)= & \frac{1}{\gamma_{\alpha ,1}!}\mathrm{per}\left(\left[F_{\alpha ,1}^{\left(\gamma_{\alpha ,1}\right)}\left(x\right)\right]\right)\\ F_{\Xi_{\alpha }}\left(x\right)= & \sum_{n=1}^{\gamma_{\alpha ,0}}\frac{1}{n!\left(\gamma_{\alpha ,0}-n\right)!}\mathrm{per}\left(\left[F_{\alpha ,0}^{\left(n\right)}\left(x\right),\;I_{\gamma_{\alpha ,0},\gamma_{\alpha ,0}-n}-F_{\alpha ,0}^{\left(\gamma_{\alpha ,0}-n\right)}\left(x\right)\right]\right). \end{array}$$

We observe that Equations (12), (13) and (14) do not depend anymore on the ill-defined product between the Dirac delta distribution and the Heaviside step function. These formulas will be used in the next subsections to calculate the mean multistability diagram of the network (Section 2.2.2), the probability that a firing-rate state is stationary for a given combination of stimuli (Section 2.2.3), and the probability that a state is stationary regardless of the stimuli (Section 2.2.4).

#### 2.2.2. Mean Multistability Diagram

The mean multistability diagram is the plot of the bifurcation points $\Lambda_{\alpha }$ and $\Xi_{\alpha }$, averaged over the network realizations. The mean bifurcation points $\langle \Lambda_{\alpha }\rangle$ and $\langle \Xi_{\alpha }\rangle$ (where the brackets $\langle \cdot \rangle$ represent the statistical mean over the network realizations) correspond to the values of the stimulus $I_{\alpha }$ at which a given firing-rate state ν loses its stability on average, turning into a different stationary state or an oscillatory solution. We propose two different approaches for evaluating the mean bifurcation points, which we implemented in the Supplemental Python script “Statistics.py”.

The first method is based on Equation (12), from which we obtain:

(15) $$\langle X\rangle =\int_{-\infty }^{+\infty }xp_{X}\left(x\right)dx=\int_{-\infty }^{+\infty }xp_{X^{c}}\left(x\right)dx+\sum_{q\in D}x_{q}\left[F_{X}\left(x_{q}\right)-\lim_{x\to x_{q}^{-}}F_{X}\left(x\right)\right],$$

for $X=\Lambda_{\alpha }$ and $X=\Xi_{\alpha }$. The cumulative distribution function $F_{X}$ in Equation (15) is calculated by means of Equation (14), while the function $p_{X^{c}}$ is given by Equation (13).

The second method takes advantage of the following formula:

$$\langle X^{z}\rangle =\int_{-\infty }^{+\infty }x^{z}dF_{X}\left(x\right)=z\int_{0}^{+\infty }x^{z-1}\left[1-F_{X}\left(x\right)+{\left(-1\right)}^{z}F_{X}\left(-x\right)\right]dx,$$

where the second equality is obtained by integrating the Lebesgue-Stieltjes integral by parts. After some algebra, in the special case z=1 we get:

(16) $$\langle X\rangle =\int_{-\infty }^{+\infty }\left[H\left(x\right)-F_{X}\left(x\right)\right]dx,$$

where $F_{X}$ is given again by Equation (14). By running both methods in the Supplemental Python script, the reader can easily check that Equations (15) and (16) provide identical results for $\langle X\rangle$, apart from rounding errors.

It is important to observe that the multistability diagram shows only those stability regions for which $\langle \Lambda_{\alpha }\rangle <\langle \Xi_{\alpha }\rangle$ for every α, because if this condition is not satisfied, the state ν is not stationary on average for any combination of stimuli. Moreover, beyond multistability, the diagram also provides a complete picture of spontaneous symmetry-breaking of the stationary solutions of the firing rates. Spontaneous symmetry-breaking occurs whenever neurons in homogeneous populations fire at different rates, despite the symmetry of the underlying neural equations. We define the population function $p\left(\cdot \right)$ that maps the neuron index $i\in \left\{0,\cdots ,N-1\right\}$ to the index α of the population the neuron *i* belongs to, so that $p\left(i\right)=\alpha$. Then, in a single network realization, a population α is said to be *homogeneous* if the sum $S_{i}^{\left(\beta \right)}\overset{\mathrm{def}}{=}\sum_{k:\;p\left(k\right)=\beta }J_{i,k}$, the firing threshold $\theta_{i}$ and the external stimulus $I_{i}$ do not depend on the index *i*, for every index *i* such that $p\left(i\right)=\alpha$ (see [28]). However, in the present article we study network statistics across realizations. For this reason, the homogeneity of a neural population should be defined in a statistical sense, namely by checking whether the probability distribution of $S_{i}^{\left(\beta \right)}$ does not depend on the index *i*, for every neuron *i* in the population α. Whenever the neurons in a population show heterogeneous firing rates despite the homogeneity condition being satisfied, we say that the symmetry of that population is spontaneously broken. In order to check whether the probability distribution of $S_{i}^{\left(\beta \right)}$ is population-dependent, it is possible to calculate numerically the Kullback-Leibler divergence $D_{\mathrm{KL}}\left(S_{i}^{\left(\beta \right)}\|S_{j}^{\left(\beta \right)}\right)$ between all the pairs of neurons i,j that belong to the same population α. However, in the Supplemental script “Statistics.py”, we checked the statistical homogeneity of the neural populations in a simpler and computationally more efficient way, though our approach is less general than that based on the Kullback-Leibler divergence. Our method relies on the assumption that a small number of moments of $S_{i}^{\left(\beta \right)},\;S_{j}^{\left(\beta \right)}$, for example just the mean and the variance:

$$\begin{array}{cc} \langle S_{i}^{\left(\beta \right)}\rangle = & \sum_{k:\;p\left(k\right)=\beta }P_{i,k}\langle W_{i,k}\rangle \\ \mathrm{Var}\left(S_{i}^{\left(\beta \right)}\right)= & \sum_{k:\;p\left(k\right)=\beta }\left(P_{i,k}\langle W_{i,k}^{2}\rangle -P_{i,j}^{2}{\langle W_{i,k}\rangle }^{2}\right), \end{array}$$

are sufficient for discriminating between the probability distributions of the two random variables. In other words, we assumed that if $\langle S_{i}^{\left(\beta \right)}\rangle \neq \langle S_{j}^{\left(\beta \right)}\rangle$ and/or $\mathrm{Var}\left(S_{i}^{\left(\beta \right)}\right)\neq \mathrm{Var}\left(S_{j}^{\left(\beta \right)}\right)$, then $S_{i}^{\left(\beta \right)},\;S_{j}^{\left(\beta \right)}$ are differently distributed, and therefore the neural population α is statistically heterogeneous. Note, however, that the probability distribution of a scalar random variable with finite moments at all orders, generally is not uniquely determined by the sequence of moments. It follows that there exist (rare) cases of differently distributed random variables that share the same sequence of moments. For this reason, the moments are not always sufficient for discriminating between two probability distributions. Note also that a sufficient condition for the sequence of moments to uniquely determine the random variable is that the moment generating function has positive radius of convergence (see Theorem (30.1) in [43]).

#### 2.2.3. Occurrence Probability of the Stationary States for a Given Combination of Stimuli

In this subsection, we calculate the probability that a given firing-rate state ν is stationary, for a fixed combination of stimuli. According to Equation (4), ν is stationary for every I∈V. Since the boundaries of V (namely the functions $\Lambda_{\alpha }$ and $\Xi_{\alpha }$) are random variables, it follows that the probability that the firing-rate state ν is stationary, for a fixed combination of stimuli $\hat{I}\overset{\mathrm{def}}{=}\left({\hat{I}}_{0},\cdots ,{\hat{I}}_{P-1}\right)$, is $P\left(\hat{I}\in V=V_{0}\times \cdots \times V_{P-1}\right)$. Since $\Lambda_{\alpha }$, given the firing rate ν and for $\alpha \in \left\{0,\cdots ,P-1\right\}$, are conditionally independent variables (and the same for the variables $\Xi_{\alpha }$), it follows that $P\left(\hat{I}\in V\right)$ can be factored out into the product of the probabilities $P\left({\hat{I}}_{\alpha }\in V_{\alpha }\right)$. In particular, whenever $\Gamma_{I_{\alpha },1}=\emptyset$, from Equation (4) we see that ν is stationary for every $I_{\alpha }<\Xi_{\alpha }$. It follows that, in this case, $P\left({\hat{I}}_{\alpha }\in V_{\alpha }\right)=P\left({\hat{I}}_{\alpha }<\Xi_{\alpha }\right)=\int_{{\hat{I}}_{\alpha }}^{+\infty }p_{\Xi_{\alpha }}\left(x\right)dx=1-F_{\Xi_{\alpha }}\left({\hat{I}}_{\alpha }\right)$. On the other hand, whenever $\Gamma_{I_{\alpha },0}=\emptyset$, the state ν is stationary for every $I_{\alpha }\geq \Lambda_{\alpha }$, so that $P\left({\hat{I}}_{\alpha }\in V_{\alpha }\right)=P\left({\hat{I}}_{\alpha }\geq \Lambda_{\alpha }\right)=\int_{-\infty }^{{\hat{I}}_{\alpha }}p_{\Lambda_{\alpha }}\left(x\right)dx=F_{\Lambda_{\alpha }}\left({\hat{I}}_{\alpha }\right)$. In all the other cases, ν is stationary for every $\Lambda_{\alpha }\leq I_{\alpha }<\Xi_{\alpha }$. This condition can be decomposed as $\left(I_{\alpha }\geq \Lambda_{\alpha }\right)\wedge \left(I_{\alpha }<\Xi_{\alpha }\right)$, and since $\Gamma_{I_{\alpha },0}\cap \Gamma_{I_{\alpha },1}=\emptyset$, the random variables $\Lambda_{\alpha }$ e $\Xi_{\alpha }$ are conditionally independent given ν, so that $P\left({\hat{I}}_{\alpha }\in V_{\alpha }\right)=P\left({\hat{I}}_{\alpha }\geq \Lambda_{\alpha }\right)P\left({\hat{I}}_{\alpha }<\Xi_{\alpha }\right)=F_{\Lambda_{\alpha }}\left({\hat{I}}_{\alpha }\right)\left[1-F_{\Xi_{\alpha }}\left({\hat{I}}_{\alpha }\right)\right]$. Therefore, to summarize, the probability that the firing-rate state ν is stationary, for a fixed combination of stimuli $\hat{I}$, is:

(17) $$\begin{array}{c} P\left(\hat{I}\in V\right)=\prod_{\alpha =0}^{P-1}P\left({\hat{I}}_{\alpha }\in V_{\alpha }\right)\\ P\left({\hat{I}}_{\alpha }\in V_{\alpha }\right)=\left\{\begin{array}{cc} 1-F_{\Xi_{\alpha }}\left({\hat{I}}_{\alpha }\right), & \mathrm{if}\;\;\Gamma_{I_{\alpha },1}=\emptyset \\ F_{\Lambda_{\alpha }}\left({\hat{I}}_{\alpha }\right), & \mathrm{if}\;\;\Gamma_{I_{\alpha },0}=\emptyset \\ F_{\Lambda_{\alpha }}\left({\hat{I}}_{\alpha }\right)\left[1-F_{\Xi_{\alpha }}\left({\hat{I}}_{\alpha }\right)\right], & \mathrm{otherwise}. \end{array}\right. \end{array}$$

Please note that $P\left(\hat{I}\in V\right)$ can be equivalently interpreted as the conditional probability $P\left(\nu |\nu ,\hat{I}\right)$ (therefore generally $\sum_{\nu \in {\left\{0,1\right\}}^{N}}P\left(\nu |\nu ,\hat{I}\right)\neq 1$, namely $P\left(\hat{I}\in V\right)$ is not normalized over the set of the possible $2^{N}$ firing-rate states.). In other terms, $P\left(\hat{I}\in V\right)$ represents the probability (across the realizations of the synaptic connectivity matrix) that, given the specific firing-rate vector ν is the initial condition of Equations (1) or (3), the network state will remain ν at the next time instants (i.e., ν is stationary). $P\left(\nu |\nu ,\hat{I}\right)$ should not be confused with the probability to observe the state ν in the long-time regime, given the initial condition of the network is distributed randomly according to some arbitrary law, across the realizations of the matrix *J*. In the latter case, similarly to spin glasses [44,45], the long-time firing rates become correlated as a consequence of the dynamics described by Equations (1) or (3), therefore the calculation of the exact probability distribution of the stationary states is expected to become intractable for an arbitrary network size.

#### 2.2.4. Occurrence Probability of the Stationary States Regardless of the Stimuli

In this subsection, we calculate the probability to observe a given firing-rate state ν in the whole multistability diagram of a single network realization, namely the probability that the state ν is stationary regardless of the specific combination of stimuli to the network. In other words, this represents the probability that ν is stationary for at least one combination of stimuli. The firing-rate state ν is observed in the multistability diagram only if its corresponding hyperrectangle V has positive hypervolume $\mathrm{vol}\left(V\right)$. Since $V=V_{0}\times \cdots \times V_{P-1}$, it follows that $\mathrm{vol}\left(V\right)=\prod_{\alpha =0}^{P-1}\mathrm{len}\left(V_{\alpha }\right)>0$ only if $\mathrm{len}\left(V_{\alpha }\right)>0\;\forall \alpha$, where $\mathrm{len}\left(V_{\alpha }\right)$ represents the length of the interval $V_{\alpha }$. Please note that in Section 3 we consider an example of neural network model with P=2, therefore in that case $\prod_{\alpha =0}^{P-1}\mathrm{len}\left(V_{\alpha }\right)$ represents the area of the rectangles in the stimuli space. Nevertheless, to avoid confusion, we will continue to use the general notation $\mathrm{vol}\left(V\right)$.

In particular, when $\Gamma_{I_{\alpha },0}=\emptyset$ (respectively $\Gamma_{I_{\alpha },1}=\emptyset$), from Equation (4) we get $\mathrm{len}\left(V_{\alpha }\right)=\infty$ for every $\Lambda_{\alpha }$ (respectively $\Xi_{\alpha }$), or in other words $P\left(\mathrm{len}\left(V_{\alpha }\right)>0\right)=1$. On the other hand, when $\Gamma_{I_{\alpha },0},\;\Gamma_{I_{\alpha },1}\neq \emptyset$, according to Equation (4) we obtain $\mathrm{len}\left(V_{\alpha }\right)=\Xi_{\alpha }-\Lambda_{\alpha }$. Since $\Lambda_{\alpha }$ and $\Xi_{\alpha }$ are conditionally independent given the firing rate ν, we can write:

$$p_{\mathrm{len}\left(V_{\alpha }\right)}\left(x\right)=\int_{-\infty }^{+\infty }p_{\Xi_{\alpha }}\left(z\right)p_{\Lambda_{\alpha }}\left(z-x\right)dz,$$

and therefore, by using Equation (12):

$$\begin{array}{cc} P\left(\mathrm{len}\left(V_{\alpha }\right)>0\right) & =\int_{0}^{+\infty }p_{\mathrm{len}\left(V_{\alpha }\right)}\left(x\right)dx=\int_{-\infty }^{+\infty }p_{\Xi_{\alpha }}\left(x\right)F_{\Lambda_{\alpha }}\left(x\right)dx\\ & =\int_{-\infty }^{+\infty }p_{\Xi_{\alpha }^{c}}\left(x\right)F_{\Lambda_{\alpha }}\left(x\right)dx+\sum_{q\in \Gamma_{I_{\alpha },0}}\left[F_{\Xi_{\alpha }}\left(\theta_{q}\right)-\lim_{x\to \theta_{q}^{-}}F_{\Xi_{\alpha }}\left(x\right)\right]F_{\Lambda_{\alpha }}\left(\theta_{q}\right). \end{array}$$

To conclude, since the quantities $\mathrm{len}\left(V_{\alpha }\right)$ are conditionally independent, we obtain that the probability to observe a given firing-rate state ν in the whole multistability diagram of a single network realization is:

(18) $$P\left(\mathrm{vol}\left(V\right)>0\right)=\prod_{\alpha =0}^{P-1}P\left(\mathrm{len}\left(V_{\alpha }\right)>0\right).$$

### 2.3. The Special Case of Multi-Population Networks Composed of Statistically Homogeneous Populations

In biological networks, heterogeneity is experimentally observed between different types of synapses (e.g., excitatory vs inhibitory ones), as well as within a given synaptic type [1]. For this reason, in this subsection we focus our attention on the study of random networks composed of P statistically homogeneous populations. As we explained in Section 2.2.2, by statistical homogeneity we mean that the synaptic weights are random and therefore heterogeneous, but the probability distribution of $S_{i}^{\left(\beta \right)}\overset{\mathrm{def}}{=}\sum_{k:\;p\left(k\right)=\beta }J_{i,k}$, as well as the firing threshold $\theta_{i}$ and the external stimulus $I_{i}$, are population-dependent. In other words, in statistically homogeneous networks the probability distribution of their connections depends only on the pre- and post-synaptic population indexes and not on the individual synaptic pair indexes, and similarly the firing thresholds and the external stimuli depend only on the population index and not on the individual neuron index. This model has been used previously in neuroscience to study the dynamical consequences of heterogeneous synaptic connections in multi-population networks (see e.g., [16,19]). However, while previous studies focused on the thermodynamic limit of the network model, here we consider the case of arbitrary-size networks.

We call $N_{\alpha }$ the size of population α, so that $\sum_{\alpha =0}^{P-1}N_{\alpha }=N$. Moreover, we rearrange the neurons so that the connection probabilities can be written in the following block-matrix form:

(19) $$P=\left[\begin{array}{cccc} P_{0,0} & P_{0,1} & \cdots & P_{0,P-1}\\ P_{1,0} & P_{1,1} & \cdots & P_{1,P-1}\\ \vdots & \vdots & \ddots & \vdots \\ P_{P-1,0} & P_{P-1,1} & \cdots & P_{P-1,P-1} \end{array}\right],\;P_{\alpha ,\beta }=\left\{\begin{array}{cc} P_{\alpha }^{\mathrm{aut}}{\mathrm{Id}}_{N_{\alpha }}+P_{\alpha ,\alpha }\left(I_{N_{\alpha }}-{\mathrm{Id}}_{N_{\alpha }}\right), & \;\mathrm{if}\;\alpha =\beta \\ P_{\alpha ,\beta }I_{N_{\alpha },N_{\beta }}, & \;\mathrm{if}\;\alpha \neq \beta . \end{array}\right.$$

In Equation (19), $P_{\alpha ,\beta }$ is a $N_{\alpha }\times N_{\beta }$ matrix, while $P_{\alpha }^{\mathrm{aut}}$ represents the magnitude of the diagonal entries of the matrix $P_{\alpha ,\alpha }$, namely the probability to observe a self-connection or autapse [46]. $P_{\alpha ,\beta }$ represents the probability to observe a synaptic connection from a neuron in population β to a (distinct) neuron in population α. Moreover, $I_{N_{\alpha },N_{\beta }}$ is the $N_{\alpha }\times N_{\beta }$ all-ones matrix (here we use the simplified notation $I_{N_{\alpha }}\overset{\mathrm{def}}{=}I_{N_{\alpha },N_{\alpha }}$), while ${\mathrm{Id}}_{N_{\alpha }}$ is the $N_{\alpha }\times N_{\alpha }$ identity matrix. According to the homogeneity assumption, we also suppose that the strength of the non-zero synaptic connections from population β to population α is generated from a population-dependent probability distribution:

$$p_{W_{i,j}}=\left\{\begin{array}{cc} p_{\alpha }^{\left(\mathrm{aut}\right)} & \mathrm{for}\;\;i=j\\ p_{\alpha ,\beta } & \mathrm{otherwise}, \end{array}\right.$$

∀i,j belonging to populations α,β, respectively. For every excitatory population the support of the distribution $p_{W_{i,j}}$ must be non-negative, while for every inhibitory population it must be non-positive. We also suppose that all the neurons in population α have the same firing threshold $\theta_{i}=\vartheta_{\alpha }$ and share the same stimulus $I_{i}=I_{\alpha }$. For example, if each population receives a distinct external stimulus, then $\Gamma_{I_{\alpha }}=\left\{n_{\alpha -1},n_{\alpha -1}+1,\cdots ,n_{\alpha }-1\right\}$, where $n_{\alpha -1}\overset{\mathrm{def}}{=}\sum_{\beta =0}^{\alpha -1}N_{\beta }$ and $n_{-1}\overset{\mathrm{def}}{=}0$. However, generally, there may exist distinct populations that share the same stimulus.

Now consider the following N×N block matrix:

$$B\overset{\mathrm{def}}{=}\left[\begin{array}{cccc} B_{0,0} & B_{0,1} & \cdots & B_{0,Y-1}\\ B_{1,0} & B_{1,1} & \cdots & B_{1,Y-1}\\ \vdots & \vdots & \ddots & \vdots \\ B_{X-1,0} & B_{X-1,1} & \cdots & B_{X-1,Y-1} \end{array}\right],$$

with homogeneous $X_{\lambda }\times Y_{\mu }$ blocks $B_{\lambda ,\mu }=B_{\lambda ,\mu }I_{X_{\lambda },Y_{\mu }}$ (where $\sum_{\lambda =0}^{X-1}X_{\lambda }=\sum_{\mu =0}^{Y-1}Y_{\mu }=N$, while $B_{\lambda ,\mu }$ are free parameters). We found that:

(20) perB=C∑s∈STsUsS=defs=s0,0,⋯,sX−1,Y−1∈NXY:∑μ=0Y−1sλ,μ=Xλ∀λ,∑λ=0X−1sλ,μ=Yμ∀μ,N=def0,1,2,⋯C=def∏λ=0X−1Xλ!,Ts=def∏μ=0Y−1Yμs0,μ,⋯,sX−1,μ,Us=def∏λ=0X−1∏μ=0Y−1Bλ,μsλ,μ,

with multinomial coefficients:

Yμs0,μ,⋯,sX−1,μ=defYμ!s0,μ!⋯sX−1,μ!.

As a consequence of the statistical homogeneity of the multi-population network considered in this subsection, the matrices in Equations (13) and (14) are composed of homogeneous block submatrices. For this reason, in the specific case of this multi-population network, the permanents in Equations (13) and (14) can be calculated by means of Equation (20). Note that, for a given α, the parameter X represents the number of distinct populations that share the current $I_{\alpha }$ (for example, X=1 if each population receives a distinct external stimulus), while Y=1,2 is the number of block columns (for example, Y=1 when calculating $F_{\Lambda_{\alpha }}\left(x\right)$, see Equation (14), and Y=2 when calculating $p_{\Lambda_{\alpha }^{c}}\left(x\right)$, see Equation (13)). $X_{\lambda }$ corresponds to the number of neurons with index $i\in \Gamma_{I_{\alpha },u}$ that belong to the population λ, while $Y_{\mu }$ represents the number of columns of the μth block column (for example, when calculating $p_{\Lambda_{\alpha }^{c}}\left(x\right)$, we set $Y_{0}=1$ and $Y_{1}=\gamma_{\alpha ,1}-1$, which correspond to the number of columns of the submatrices $a_{\alpha ,1}\left(\theta -x\right)$ and $F_{\alpha ,1}^{\left(\gamma_{\alpha ,1}-1\right)}\left(x\right)$ respectively, see Equation (13)). Moreover, N corresponds to $\gamma_{\alpha ,u}$, while the parameters $B_{\lambda ,\mu }$ represent the entries of the matrices in Equations (13) and (14) (for example, $N=\gamma_{\alpha ,1}$, $B_{\lambda ,0}=a_{i}\left(\theta_{i}-x\right)$ and $B_{\lambda ,1}=F_{I_{i}}\left(x\right)$ for *i* in the population λ, when calculating $p_{\Lambda_{\alpha }^{c}}\left(x\right)$).

For the sake of clarity, we implemented Equation (20) in the Supplemental Python script “Permanent.py”. Since the permanents in Equations (13) and (14) can be obtained from Equation (20) for Y=1 and Y=2, in the script we specifically implemented these two cases. The computation of $\mathrm{per}\left(B\right)$ by means of Equation (20) generally prove much faster than the BBFG algorithm, see Section 3. However, it is important to note that while the BBFG algorithm can be applied to neural networks with any topology, Equation (20) is specific for multi-population networks composed of statistically homogeneous populations.

### 2.4. Large-Network Limit

In computational neuroscience, statistically homogeneous multi-population networks represent an important class of network models, since their large-size limit is typically well-defined and serves as a basis for understanding the asymptotic behavior of neural systems [16,17,18,19,20]. In this subsection, we derive the large-size limit of the class of statistically homogeneous multi-population networks with quenched disorder that we introduced in Section 2.3. In particular, we focus on the case when each neural population receives a distinct external stimulus current, and we also suppose that the contribution of self-connections to the statistics of the firing rates is negligible in the large-network limit. The consequences of the relaxation of these two assumptions will be discussed at the end of this subsection.

The derivation of the asymptotic form of the stationary-state statistics requires the introduction of a proper normalization of the sum $S_{i}=\sum_{j=0}^{N-1}J_{i,j}\nu_{j}$ in Equation (1), in order to prevent the divergence of the mean and the variance of $S_{i}$ in the thermodynamic limit. For this purpose, we choose the mean and the variance of the random variables $W_{i,j}$ as follows:

(21) $$\begin{array}{c} m_{\alpha ,\beta }\overset{\mathrm{def}}{=}\langle W_{i,j}\rangle =\frac{\mu_{\alpha ,\beta }}{N_{\beta }}\\ s_{\alpha ,\beta }^{2}\overset{\mathrm{def}}{=}\mathrm{Var}\left(W_{i,j}\right)=\frac{\sigma_{\alpha ,\beta }^{2}}{N_{\beta }}+{\left(\frac{\mu_{\alpha ,\beta }}{N_{\beta }}\right)}^{2}\left(P_{\alpha ,\beta }-1\right), \end{array}$$

given parameters $\mu_{\alpha ,\beta }$, $\sigma_{\alpha ,\beta }$, $N_{\beta }$ and $P_{\alpha ,\beta }$ such that $\mu_{\alpha ,\beta }\in R$ and $\mathrm{Var}\left(W_{i,j}\right)\in R_{\geq 0}$, for every i,j (with i≠j) in the populations α,β, respectively. Equation (21) implies that:

$$\begin{array}{c} \langle J_{i,j}\rangle =\langle T_{i,j}\rangle \langle W_{i,j}\rangle =\frac{P_{\alpha ,\beta }}{N_{\beta }}\mu_{\alpha ,\beta }\\ \mathrm{Var}\left(J_{i,j}\right)=\langle T_{i,j}^{2}\rangle \langle W_{i,j}^{2}\rangle -{\langle T_{i,j}\rangle }^{2}{\langle W_{i,j}\rangle }^{2}=\frac{P_{\alpha ,\beta }}{N_{\beta }}\sigma_{\alpha ,\beta }^{2}, \end{array}$$

and therefore:

$$\begin{array}{c} \mu_{\alpha }\overset{\mathrm{def}}{=}\langle \sum_{j=0}^{N-1}J_{i,j}\nu_{j}\rangle \approx \sum_{\beta =0}^{P-1}\frac{\gamma_{\beta ,1}P_{\alpha ,\beta }}{N_{\beta }}\mu_{\alpha ,\beta }\\ \sigma_{\alpha }^{2}\overset{\mathrm{def}}{=}\mathrm{Var}\left(\sum_{j=0}^{N-1}J_{i,j}\nu_{j}\right)\approx \sum_{\beta =0}^{P-1}\frac{\gamma_{\beta ,1}P_{\alpha ,\beta }}{N_{\beta }}\sigma_{\alpha ,\beta }^{2}, \end{array}$$

having neglected the contribution of the autapses. Therefore the mean and the variance of $S_{i}$ are finite for every state ν in the thermodynamic limit, as desired. Now, consider any of the firing-rate states ν composed of $\gamma_{\alpha ,1}$ active neurons in the population α ($\forall \alpha \in \left\{0,\cdots ,P-1\right\}$). For the central limit theorem, given any distribution (not necessarily normal) of $W_{i,j}$ that satisfies Equation (21), we get:

$$\sqrt{\gamma_{\beta ,1}}\left(\frac{1}{\gamma_{\beta ,1}}\sum_{j=n_{\beta -1}}^{n_{\beta }-1}J_{i,j}\nu_{j}-\frac{P_{\alpha ,\beta }}{N_{\beta }}\mu_{\alpha ,\beta }\right)\overset{d}{\to }N\left(0,\frac{P_{\alpha ,\beta }}{N_{\beta }}\sigma_{\alpha ,\beta }^{2}\right)\;\forall \beta ,$$

in the limit $\gamma_{\beta ,1}\to \infty$ (see Section 2.3 for the definition of the parameter $n_{\beta }$). In turn, this implies that:

$$I_{i}=\theta_{i}-\sum_{\beta =0}^{P-1}\sum_{j=n_{\beta -1}}^{n_{\beta }-1}J_{i,j}\nu_{j}\overset{d}{\to }N\left(\vartheta_{\alpha }-\mu_{\alpha },\sigma_{\alpha }^{2}\right).$$

Since the random variables $I_{i}$ are conditionally independent given ν and identically distributed $\forall i\in \Gamma_{I_{\alpha }}$ and α fixed, according to the *Fisher-Tippett-Gnedenko theorem* [47,48,49], the distribution of the variables $\Lambda_{\alpha }$ and $\Xi_{\alpha }$ converges to the *Gumbel distribution* in the limit $\gamma_{\alpha ,u}\to \infty$. In other words, given $X\in \left\{\Lambda ,\Xi \right\}$, and by defining, according to [50]:

(22) $$\begin{array}{c} z_{X_{\alpha }}\left(x\right)\overset{\mathrm{def}}{=}\frac{x-a_{X_{\alpha }}}{b_{X_{\alpha }}}\\ a_{\Lambda_{\alpha }}\overset{\mathrm{def}}{=}\vartheta_{\alpha }-\mu_{\alpha }+\sigma_{\alpha }\Phi^{-1}\left(1-\frac{1}{n_{\Lambda_{\alpha }}}\right),\;a_{\Xi_{\alpha }}\overset{\mathrm{def}}{=}\vartheta_{\alpha }-\mu_{\alpha }-\sigma_{\alpha }\Phi^{-1}\left(1-\frac{1}{n_{\Xi_{\alpha }}}\right)\\ b_{X_{\alpha }}\overset{\mathrm{def}}{=}\sigma_{\alpha }\left[\Phi^{-1}\left(1-\frac{1}{n_{X_{\alpha }}e}\right)-\Phi^{-1}\left(1-\frac{1}{n_{X_{\alpha }}}\right)\right]\\ n_{\Lambda_{\alpha }}\overset{\mathrm{def}}{=}\gamma_{\alpha ,1},\;n_{\Xi_{\alpha }}\overset{\mathrm{def}}{=}\gamma_{\alpha ,0}, \end{array}$$

then in the limit $n_{X_{\alpha }}\to \infty$ we get:

(23) $$\begin{array}{c} p_{\Lambda_{\alpha }}\left(x\right)\to g\left(z_{\Lambda_{\alpha }}\left(x\right)\right),\;F_{\Lambda_{\alpha }}\left(x\right)\to G\left(z_{\Lambda_{\alpha }}\left(x\right)\right)\\ p_{\Xi_{\alpha }}\left(x\right)\to g\left(-z_{\Xi_{\alpha }}\left(x\right)\right),\;F_{\Xi_{\alpha }}\left(x\right)\to 1-G\left(-z_{\Xi_{\alpha }}\left(x\right)\right), \end{array}$$

where $g\left(\cdot \right)$ and $G\left(\cdot \right)$ are the Gumbel probability density and its cumulative distribution function, respectively:

$$g\left(x\right)=\frac{1}{b_{X_{\alpha }}}e^{-\left(x+e^{-x}\right)},\;G\left(x\right)=e^{-e^{-x}}.$$

In Equation (22), $\Phi \left(\cdot \right)$ represents the cumulative distribution function of the standard normal probability density. $\Phi^{-1}\left(\cdot \right)$ is the *probit function*, which can be expressed in terms of the inverse of the error function $\mathrm{erf}\left(\cdot \right)$ as $\Phi^{-1}\left(x\right)=\sqrt{2}{\mathrm{erf}}^{-1}\left(2x-1\right)$. By using an asymptotic expansion of ${\mathrm{erf}}^{-1}\left(\cdot \right)$, we get:

$$\begin{array}{cc} \Phi^{-1}\left(1-\frac{1}{n_{X_{\alpha }}}\right)\approx & \sqrt{\ln\left(\frac{n_{X_{\alpha }}^{2}}{2\pi }\right)-\ln\left(\ln\left(\frac{n_{X_{\alpha }}^{2}}{2\pi }\right)\right)}\\ \Phi^{-1}\left(1-\frac{1}{n_{X_{\alpha }}e}\right)\approx & \sqrt{2+\ln\left(\frac{n_{X_{\alpha }}^{2}}{2\pi }\right)-\ln\left(2+\ln\left(\frac{n_{X_{\alpha }}^{2}}{2\pi }\right)\right)}. \end{array}$$

Moreover, it is possible to prove that:

(24) $$\langle \Lambda_{\alpha }\rangle =a_{\Lambda_{\alpha }}+b_{\Lambda_{\alpha }}\gamma ,\;\langle \Xi_{\alpha }\rangle =a_{\Xi_{\alpha }}-b_{\Xi_{\alpha }}\gamma ,$$

where γ is the Euler-Mascheroni constant. Please note that Equation (24) can be used for plotting the mean multistability diagram of the network, while Equations (17), (22) and (23) provide an analytical expression of the occurrence probability of the stationary states for a given combination of stimuli. Unfortunately, we are not aware of any exact formula of the occurrence probability of the stationary states regardless of the stimuli (see Section 2.2.4). For this reason, the latter should be calculated numerically or through analytical approximations, from Equation (18).

Now we discuss the two assumptions that we made in the derivation of our results, namely distinct external stimuli to each neural population, and a negligible contribution of the autapses to the statistics of the firing rates. The relaxation of the first assumption implies the calculation of the minimum and maximum of non-identically distributed random variables. For example, in the case when an external stimulus is shared by two distinct populations, the variable $I_{i}$ has two distinct probability distributions, depending on the population the neuron *i* belongs to. However, the Fisher-Tippett-Gnedenko theorem is valid only for identically distributed variables, and a straightforward generalization of the theorem to the case of non-identically distributed variables is not available (see Section 4.2). Note, however, that this limitation applies only to the asymptotic expansion discussed in the present subsection. The exact (i.e., non-asymptotic) theory discussed in Section 2.2 is not affected by this limitation, and is also valid when a stimulus is shared by several populations.

The second assumption in our derivation was the negligible contribution of the autapses to the statistics of the firing rates in the large-network limit. This assumption can be relaxed for example by supposing that the autapses are not scaled, so that the random variable $S_{i}$ is strongly affected by the autaptic weight $J_{i,i}$ when $\nu_{i}=1$. In this case, the central limit theorem does not apply anymore to the whole sum $S_{i}=\sum_{j=0}^{N-1}J_{i,j}\nu_{j}$, but only to $S_{i}-J_{i,i}$. In other words, in the case when the autapses are not normally distributed, the sum $S_{i}$ is not normally distributed either, therefore the distribution of the variables $\Lambda_{\alpha }$ and $\Xi_{\alpha }$ may not be necessarily the Gumbel law. This case can be studied analytically, if desired, but we omitted it for the sake of brevity.

### 2.5. Numerical Simulations

**Figure 1:**
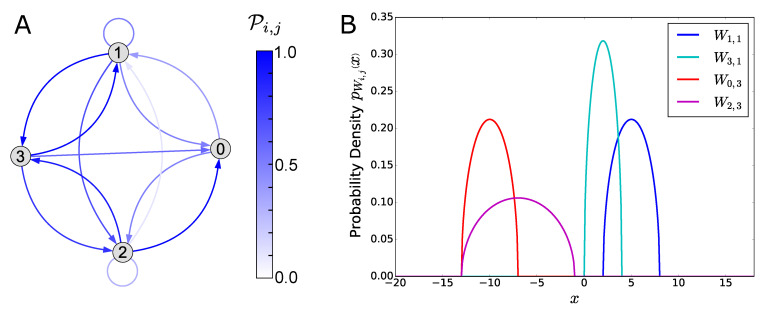
Probability distribution of the synaptic weights. This figure shows the probability distribution of the synaptic weights $J_{i,j}$ (see Equation (2)), in the specific case of the small-size network model described in Section 3. (**A**) Graph of the connection probability matrix P reported in Table 1. An arrow from the vertex *j* to the vertex *i* represents a connection probability $P_{i,j}>0$. (**B**) Wigner semicircle distribution of some variables $W_{i,j}$, according to Equation (25) and the values of the parameters C and R reported in Table 1.

**Figure 2:**
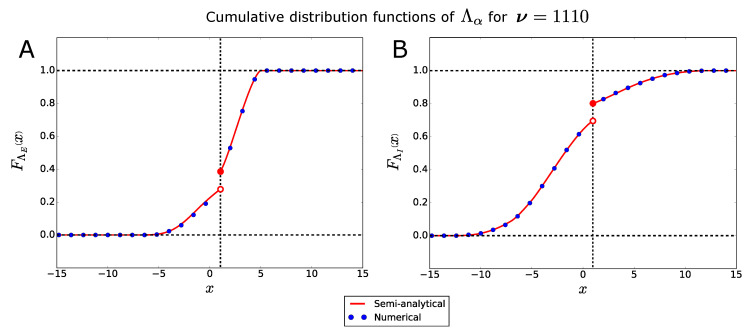
Examples of cumulative distribution functions of the bifurcation points. This figure reports the cumulative distribution functions of the bifurcation points $\Lambda_{E}$ and $\Lambda_{I}$ of the firing-rate state 1110 (panels (**A**) and (**B**), respectively), in the case of the small-size network described in Section 3. The red curves represent the semi-analytical functions, calculated through Equation (14), while the blue dots represent the numerical functions, computed over 5000 network realizations as described in Section 2.5. Similar results can be derived for all the other bifurcation points of the network, if desired.

**Figure 3:**
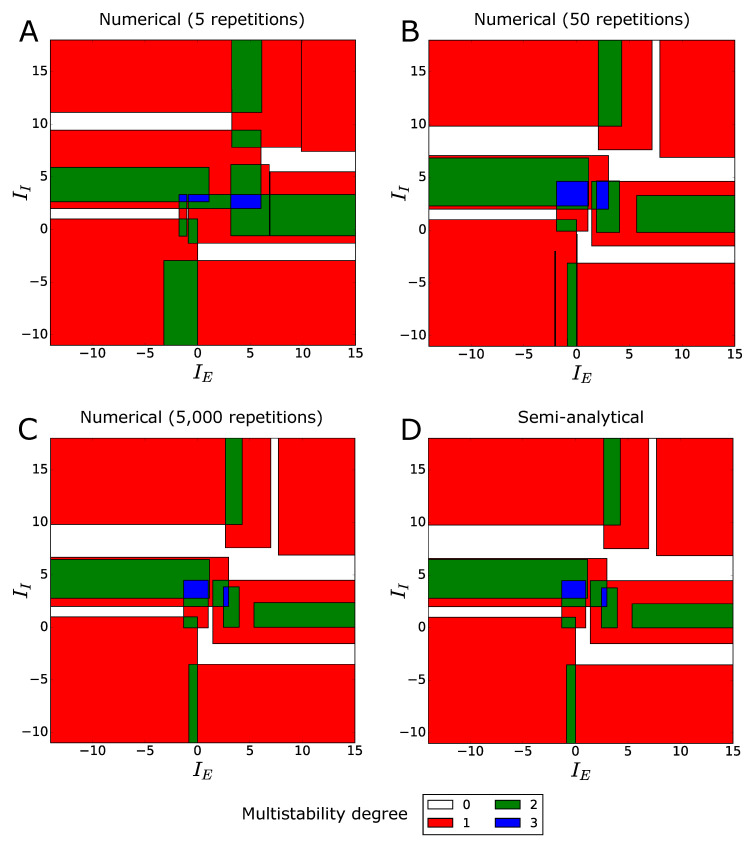
Mean multistability diagram. This figure reports the mean multistability diagram of the small-size network described in Section 3, see Equation (25) and Table 1. The diagram shows how the degree of multistability of the network, namely the number of stationary solutions, depends on average on the external currents $I_{E}$ and $I_{I}$. Each color represents a different degree of multistability M (e.g., blue = tristability). (**A**–**C**) Numerical multistability diagrams, obtained through Monte Carlo simulations as described in Section 2.5, for an increasing number of network realizations (5, 50 and 5000). (**D**) Semi-analytical multistability diagram, obtained through the techniques described in Section 2.2.2. Please note that by increasing the number of network realizations, the numerical multistability diagram converges to the semi-analytical one.

**Figure 4:**
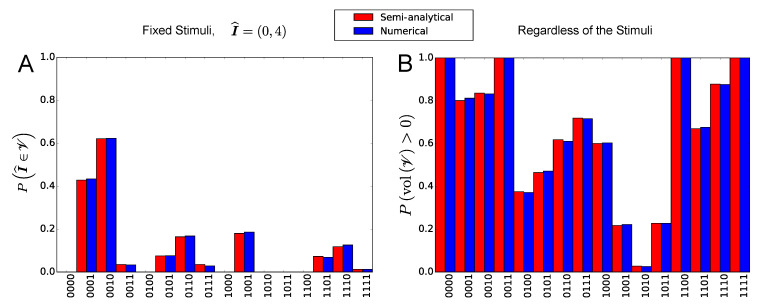
Occurrence probability of the firing-rate states. This figure reports the occurrence probability of the $2^{N}=16$ firing-rate states of the network described in Section 3 (see Equation (25) and Table 1), from the state 0000 to the state 1111. (**A**) Occurrence probability of the firing-rate states for fixed stimuli, obtained for ${\hat{I}}_{E}=0$ and ${\hat{I}}_{I}=4$. The red bars represent the occurrence probability calculated semi-analytically through the method described in Section 2.2.3. The blue bars represent the same probability, evaluated numerically by a Monte Carlo simulation, as explained in Section 2.5; (**B**) Occurrence probability of the firing-rate states, regardless of the stimuli (red bars calculated according to the approach of Section 2.2.4, blue bars computed again through a Monte Carlo simulation). In both panels, we computed the blue bars over 5000 network realizations. Please note that the occurrence probabilities are not normalized over the set of $2^{N}$ firing-rate states, see text.

To further validate our results, in Section 3 we compare our semi-analytical formulas with numerical Monte Carlo simulations, that we implemented in the “Numerical Simulations” section of the Supplemental Python script “Statistics.py”. During these numerical simulations, we run 5000 realizations of the network described in Figure 1 and Table 1, obtaining the numerical results shown in Figure 2, Figure 3 and Figure 4. At each network realization, we generate a new (quenched) connectivity matrix *J*, according to Equation (8). Then, for each *J*, we derive the corresponding bifurcation points $\Lambda_{\alpha }$ and $\Xi_{\alpha }$ and the hyperrectangles V, by applying the algorithm “Multistability_Diagram.py” that we introduced in [28].

The cumulative distribution function of the bifurcation points is then computed by means of a cumulative sum of the probability histograms of $\Lambda_{\alpha }$ and $\Xi_{\alpha }$. This provides a numerical approximation of the functions $F_{\Lambda_{\alpha }}\left(x\right)$ and $F_{\Xi_{\alpha }}\left(x\right)$, that we derived semi-analytically in Equation (14).

The mean multistability diagram is calculated by averaging the bifurcation points $\Lambda_{\alpha }$ and $\Xi_{\alpha }$ over the network realizations. This provides a numerical approximation of the quantities $\langle \Lambda_{\alpha }\rangle$ and $\langle \Xi_{\alpha }\rangle$, that we derived semi-analytically in Equations (15) and (16).

The probability that a given firing-rate state ν is stationary for a fixed combination of stimuli $\hat{I}$ is calculated by counting, during the Monte Carlo simulations, the number of times $\hat{I}\in V$. By dividing this number by the total number of realizations, we obtain a numerical estimation of the probability $P\left(\hat{I}\in V\right)$ (see Equation (17) for its semi-analytical expression). This calculation is then repeated for each of the $2^{N}$ firing-rate states ν. Alternatively, this probability can be calculated by counting the relative number of times $\nu \left(t_{0}+1\right)=\nu \left(t_{0}\right)$ (stationarity condition), where the firing-rate state $\nu \left(t_{0}\right)$ is each of the $2^{N}$ initial conditions of the network model, while the state $\nu \left(t_{0}+1\right)$ is calculated iteratively from it by means of Equation (1) or Equation (3). We implemented both methods in the Python script “Statistics.py”, and the reader can easily check that they provide identical numerical estimations of $P\left(\hat{I}\in V\right)$.

The probability that the state ν is stationary, regardless of the specific combination of stimuli to the network, is derived numerically by counting the relative number of times $\mathrm{vol}\left(V\right)=\prod_{\alpha =0}^{P-1}\mathrm{len}\left(V_{\alpha }\right)>0$, for each of the $2^{N}$ firing-rate states ν. This provides a numerical estimation of the probability $P\left(\mathrm{vol}\left(V\right)>0\right)$, that we derived semi-analytically in Equation (18).

**Figure 5:**
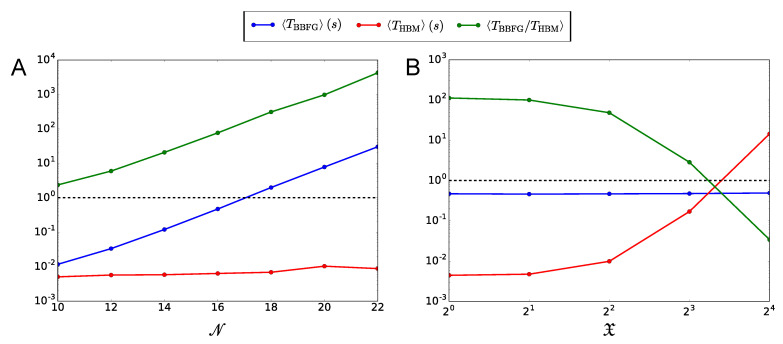
Speed test for the analytical formula of the permanent. This figure shows the mean computational times $\langle T_{\mathrm{BBFG}}\rangle$ and $\langle T_{\mathrm{HBM}}\rangle$ (see text) required for calculating the permanent of homogeneous block matrices B by means of an Intel^®^ Core™ i5-5300U CPU clocked at 2.30 GHz with 16 GB RAM. We chose the entries of the matrices B to be independent random numbers $0\leq B_{\lambda ,\mu }<0.3$ with two decimal digits, generated from a uniform probability distribution (see the Python script “Permanent.py”), while the remaining parameters of B are shown in Table 2. The average times $\langle T_{\mathrm{BBFG}}\rangle$ and $\langle T_{\mathrm{HBM}}\rangle$ are calculated over 100 repetitions of the matrices. (**A**) Mean computational times $\langle T_{\mathrm{BBFG}}\rangle$ (blue line) and $\langle T_{\mathrm{HBM}}\rangle$ (red line) in seconds, as a function of N. The green line represents the mean speed gain $\langle T_{\mathrm{BBFG}}/T_{\mathrm{HBM}}\rangle$ of Equation (20) over the BBFG algorithm. (**B**) Mean computational times and speed gain as a function of X.

Then, in Figure 5 we show a speed test of Equation (20), for the calculation of the permanent of a block matrix with homogeneous blocks described in Table 2. The computational time required by Equation (20) is compared with the time required by the Balasubramanian-Bax-Franklin-Glynn algorithm [39,40,41,42].

**Figure 6:**
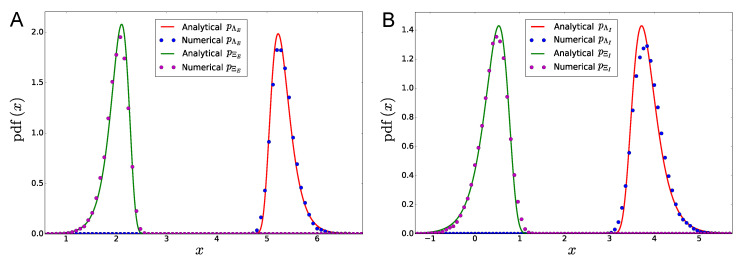
Large-size limit of a statistically homogeneous two-population network. This figure shows the probability distribution of the bifurcation points in a random network composed of two statistically homogeneous populations (one excitatory and one inhibitory) with Laplace-distributed weights (see Equation (26)), in the large-size limit. The parameters of the network are reported in Table 3. In particular, note that in this figure we compute the probability density of the bifurcation points for every firing-rate state ν that is composed of $\gamma_{E,1}=240$ (respectively $\gamma_{I,1}=80$) active neurons in the excitatory (respectively inhibitory) population. The analytical curves (see the green and red solid lines) are obtained from Equations (22) and (23), while the numerical probability densities (magenta and blue dots) are calculated over 100,000 network realizations, as described in Section 2.5. (**A**) Analytical and numerical probability distributions of the the bifurcation points $\Lambda_{E}$ and $\Xi_{E}$; (**B**) Probability distributions of $\Lambda_{I}$ and $\Xi_{I}$.

To conclude, in Section 3 we run 100,000 realizations of the two-population statistically homogeneous network described in Table 3, obtaining the numerical results reported in Figure 6. The probability distribution of the bifurcation points in the large-size limit is calculated numerically through a kernel density estimation. The density estimator is applied to the samples of the random variables $\Lambda_{\alpha }$ and $\Xi_{\alpha }$, which are generated during the Monte Carlo simulations according to Equation (4), given the firing-rate state ν.

## 3. Results

In this section, we report the comparison between, on the one hand, the semi-analytical formulas of the mean multistability diagram, of the occurrence probability of the stationary firing-rate states, and of the probability distribution of the bifurcation points in both the small and large-size limits (see Section 2.2, Section 2.3 and Section 2.4), and, on the other hand, the corresponding numerical counterparts (Section 2.5).

For illustrative purposes, we consider the case P=2, so that the multistability diagram can be visualized on a plane. In particular, we suppose that the network is composed of excitatory (*E*) and inhibitory (*I*) neurons. For this reason, it is convenient to change the notation slightly, and to consider $\alpha \in \left\{E,I\right\}$ rather than $\alpha \in \left\{0,1\right\}$ (so that the multistability diagram will be plotted on the $I_{E}-I_{I}$ plane). Since the total number of firing-rate states of the network increases as $2^{N}$ with the network size, in this section we apply the Python script “Statistics.py” to a small-sized network (N=4), in order to ensure the clarity of the figures. It is important to note that, in the derivation of the results in Section 2.2 and Section 2.3, we did not resort to any mathematical approximation, and that our semi-analytical formulas are exact for every size *N*. For this reason, if desired, our script can be applied to networks with size N≫4, depending on the computational power available.

In the network that we test, we suppose that the neurons with indexes $i\in \left\{0,1\right\}$ are excitatory and receive an external stimulus $I_{E}$, while the neurons with indexes $i\in \left\{2,3\right\}$ are inhibitory and receive a stimulus $I_{I}$. Moreover, we suppose that the independent random variables $W_{i,j}$ are distributed according to the following Wigner semicircle distribution:

(25) $$p_{W_{i,j}}\left(x\right)=\left\{\begin{array}{cc} \frac{2}{\pi R_{i,j}^{2}}\sqrt{R_{i,j}^{2}-{\left(x-C_{i,j}\right)}^{2}}, & \mathrm{if}\;\;\left|x-C_{i,j}\right|\leq R_{i,j}\\ 0, & \mathrm{otherwise}, \end{array}\right.$$

centered at $x=C_{i,j}$ and with radius $R_{i,j}$. In Table 1 we report the values of the parameters P, $\theta ={\left[\theta_{0},\;\theta_{1},\;\theta_{2},\;\theta_{3}\right]}^{T}$, C and R that we chose for this network.

In panel A of Figure 1 we show the graph of the connection probability matrix P, while in panel B we plot some examples of the Wigner probability distributions $p_{W_{i,j}}$.

Please note that for our choice of the parameters, the support of the Wigner distribution, namely the range $\left[C_{i,j}-R_{i,j},C_{i,j}+R_{i,j}\right]$, is a subset of $R_{\geq 0}$ (respectively $R_{\leq 0}$) for excitatory (respectively inhibitory) neurons. It follows that the connectivity matrix *J* of the model satisfies the Dale’s principle [51], as required for biologically realistic networks (note, however, that our algorithm can also be applied to networks that do not satisfy the principle, if desired).

In Figure 2 we plot the cumulative distribution functions of the bifurcation points $\Lambda_{E}$ and $\Lambda_{I}$ of, e.g., the firing-rate state 1110.

The figure shows a very good agreement between the semi-analytical functions (red curves), calculated through Equation (14), and the numerical functions (blue dots), computed over 5000 network realizations as described in Section 2.5. Please note that, generally, the cumulative distribution functions are not continuous (see also Section 2.2.1), and that jump discontinuities may occur at the firing thresholds (θ=1, in this example).

In Figure 3 we plot the comparison between the mean multistability diagram of the network, evaluated numerically through 5, 50 and 5000 Monte-Carlo repetitions (panels A–C), and the same diagram, evaluated semi-analytically through Equation (15) or Equation (16) (panel D).

The figure shows that, by increasing the number of network realizations, the numerical multistability diagram converges to the semi-analytical one (compare panels C and D), apart from small numerical errors, that depend on the integration step in the semi-analytical formulas, and on the finite number of repetitions in the Monte-Carlo simulations. The diagrams show a complex pattern of multistability areas in the $I_{E}-I_{I}$ plane, characterized by multistability degrees M=1 (monostability), 2 (bistability), and 3 (tristability). A similar result was already observed in small binary networks with deterministic synaptic weights, see [28,52]. Moreover, note that our algorithm detected the presence of white areas, characterized by multistability degree M=0. In these areas, we do not observe the formation of stationary firing-rate states, so that the only possible long-time dynamics for those combinations of stimuli is represented by neural oscillations. However, generally, oscillations in the firing-rate states may also co-occur with stationary states in areas of the $I_{E}-I_{I}$ plane where M>0. The reader is referred to Section 4.3 for a discussion about the possibility to extend our work to the study of neural oscillations.

In Figure 4 we plot the comparison between the semi-analytical and numerical occurrence probability of the stationary firing-rate states (red and blue bars, respectively).

In panels A and B we plot, respectively, the probability that the state ν is stationary for a fixed combination of stimuli (${\hat{I}}_{E}=0$ and ${\hat{I}}_{I}=4$, red bars calculated through Equation (17)), and the probability that ν is stationary regardless of the stimuli (red bars calculated through Equation (18)). The figure shows again a very good agreement between semi-analytical and numerical results.

In particular, panel A shows that, for the network parameters that we chose (see Table 1), the states 0000, 0100, 1000, 1010, 1011 and 1100 are never stationary for ${\hat{I}}_{E}=0$ and ${\hat{I}}_{I}=4$. In other words, in every network realization the rectangles V corresponding to these states never contain the point of coordinates $\hat{I}=\left({\hat{I}}_{E},{\hat{I}}_{I}\right)$. However, panel B shows that, at least for other combinations of the stimuli, these firing-rate states can also be stationary.

Moreover, panel B shows that the firing-rate states 0000, 0011, 1100 and 1111 have unit probability to be observed in the whole multistability diagram of a single network realization, namely $P\left(\mathrm{vol}\left(V\right)>0\right)=1$ for these states. This is a consequence of the fact that, for these states, $\Gamma_{I_{\alpha },u}=\emptyset$ for both α=E and α=I and for some *u* (for example, $\Gamma_{I_{E},1}=\Gamma_{I_{I},0}=\emptyset$ for the state 0011). For this reason, we get $\mathrm{len}\left(V_{E}\right)=\mathrm{len}\left(V_{I}\right)=\infty$, namely $P\left(\mathrm{len}\left(V_{E}\right)>0\right)=P\left(\mathrm{len}\left(V_{I}\right)>0\right)=1$, so that $P\left(\mathrm{vol}\left(V\right)>0\right)=1$ (see Section 2.2.4). On the other hand, for all the other firing-rate states, we obtain $\Gamma_{I_{\alpha },u}=\emptyset$ only for one value of the index α (for example, $\Gamma_{I_{E},1}=\emptyset$, $\Gamma_{I_{E},0}=\left\{0,1\right\}$, $\Gamma_{I_{I},1}=\left\{3\right\}$ and $\Gamma_{I_{I},0}=\left\{2\right\}$ for the state 0001), or for none of them (for example, $\Gamma_{I_{E},0}=\left\{0\right\}$, $\Gamma_{I_{E},1}=\left\{1\right\}$, $\Gamma_{I_{1},0}=\left\{2\right\}$ and $\Gamma_{I_{1},1}=\left\{3\right\}$ for the state 0101). Therefore, for these states, typically $P\left(\mathrm{vol}\left(V\right)>0\right)<1$.

In Figure 5 we show the speed gain of Equation (20) over the BBFG algorithm, achieved during the calculation of the permanent of homogeneous block matrices that we implemented in the Python script “Permanent.py”.

The matrices were generated randomly, according to the parameters in Table 2.

In particular, panel A shows that the mean computational time, that we called $\langle T_{\mathrm{BBFG}}\rangle$, required for calculating the permanent by means of the BBFG algorithm over several realizations of the matrix, increases exponentially with the matrix size N. On the contrary, the mean time required by Equation (20), that we called $\langle T_{\mathrm{HBM}}\rangle$, increases very slowly with N, resulting in a progressive and considerable improvement of performance over the BBFG algorithm (mean speed gain $\langle T_{\mathrm{BBFG}}/T_{\mathrm{HBM}}\rangle \gg 1$).

Panel B of Figure 5 shows the limitations of Equation (20). While $\langle T_{\mathrm{BBFG}}\rangle$ does not depend on the parameter X (namely the number of neural populations that share the same external stimulus), $\langle T_{\mathrm{HBM}}\rangle$ strongly increases with X, resulting in a progressive loss of performance of Equation (20) over the BBFG algorithm. This is a consequence of the increasing number of multinomial coefficients that, according to Equation (20), must be calculated in order to evaluate the matrix permanent when X is incremented. In more detail, the total number of multinomial coefficients is:

∑μ=0Y−1Yμ+X−1X−1∼∑μ=0Y−1XYμYμ!1+YμYμ+12X+OX−2,X≤∑μ=0Y−1Yμ=N,

where the asymptotic expansion holds in the limit X→∞. Our analysis shows that generally, in the study of statistically homogeneous multi-population networks, Equation (20) should be preferred to the BBFG algorithm when X≪N, namely when each stimulus is shared by a relatively small number of populations.

In Figure 6 we show examples of the probability distributions of the bifurcation points in a large random network composed of two statistically homogeneous populations (one excitatory and one inhibitory).

The network parameters that we used are reported in Table 3.

In particular, we suppose that the weights are distributed according to the following Laplace probability density:

(26) $$p_{W_{i,j}}\left(x\right)=\frac{1}{\sqrt{2s_{\alpha ,\beta }}}e^{-\sqrt{\frac{2}{s_{\alpha ,\beta }}}\left|x-m_{\alpha ,\beta }\right|},$$

∀i,j belonging to populations α,β, respectively ($m_{\alpha ,\beta }$ and $s_{\alpha ,\beta }$ are defined as in Equation (21)). Figure 6 shows a good agreement between the analytical formula of the Gumbel probability density function and numerical simulations, despite slight differences between analytical and numerical densities can be observed, as a consequence of the finite size of the network. These differences disappear in the limit N→∞. In particular, Figure 6 shows that the firing-rate states with 240 active neurons in the excitatory population, and 80 active neurons in the inhibitory one, are very unlikely to be observed in the whole multistability diagram of the network. For these states, and for our choice of the network parameters (see Table 3), the probability to get $\Xi_{E}>\Lambda_{E}$ and $\Xi_{I}>\Lambda_{I}$ is very small, as a consequence of the large distance between the peaks of the distributions of $\Lambda_{\alpha }$ and $\Xi_{\alpha }$. More generally, for a network composed of an arbitrary number P of statistically homogeneous populations, we found that the stationary states that are more likely to occur in the large-size limit are those characterized by homogeneous intra-population firing rates, namely the stationary states of the form:

(27) $$\nu =\overset{N_{0}-\mathrm{times}}{\overset{︷}{{\bar{\nu }}_{0}\cdots {\bar{\nu }}_{0}}}\overset{N_{1}-\mathrm{times}}{\overset{︷}{{\bar{\nu }}_{1}\cdots {\bar{\nu }}_{1}}}\cdots \overset{N_{P-1}-\mathrm{times}}{\overset{︷}{{\bar{\nu }}_{P-1}\cdots {\bar{\nu }}_{P-1}}},\;{\bar{\nu }}_{\alpha }\in \left\{0,1\right\},\;\forall \alpha \in \left\{0,\cdots ,P-1\right\}.$$

This result proves that, in the large-size limit, the stationary states of this network can be studied through a dimensional reduction of the model. In other words, in order to completely characterize the statistical properties of this network, it suffices to consider the firing-rate states of the form (27), since the states that present intra-population symmetry breaking are very unlikely to be observed. The main consequence of this phenomenon is a tremendous simplification in the mathematical analysis of the network model, since it reduces the analysis of the $2^{N}$ states of the network to only $2^{P}$ states of the form (27). In turn, this simplification implies a strong reduction of the computational time of the algorithms, since typically P≪N.

## 4. Discussion

We studied how the statistical properties of the stationary firing-rate states of a binary neural network model with quenched disorder depend on the probability distribution of the synaptic weights and on the external stimuli. The size of the network is arbitrary and finite, while the synaptic connections between neurons are assumed to be independent (not necessarily identically distributed) random variables, with arbitrary marginal probability distributions. By applying the results derived in [34,35,36,37] for the order statistics of sets of independent random variables, our assumptions about the network model allowed us to calculate semi-analytically the statistical properties of the stationary states and of their bifurcation points, in terms of the permanent of special matrices.

In particular, in Section 2.2.1 we derived the probability density and the cumulative distribution functions of the bifurcation points of the model in the stimuli space. From these distributions, in Section 2.2.2 we derived the mean multistability diagram of the network, namely the plot of the bifurcation points averaged over network realizations. Then, in Section 2.2.3 and Section 2.2.4, we derived the probability that a given firing-rate state is stationary for a fixed combination of stimuli, and the probability that a state is stationary regardless of the stimuli. These results provide a detailed description of the statistical properties of arbitrary-size networks with arbitrary connectivity matrix in the stationary regime, and describe how these properties are affected by variations in the external stimuli.

In Section 2.3 we specialized to the case of statistically homogeneous multi-population networks of arbitrary finite size. For these networks, we found a compact analytical formula of the permanent, which outperforms of several orders of magnitude the fastest known algorithm for the calculation of the permanent, i.e., the Balasubramanian-Bax-Franklin-Glynn algorithm [39,40,41,42]. Then, in Section 2.4 we derived asymptotic expressions of the statistical behavior of these multi-population networks in the large-size limit. In particular, if the contribution of the autapses to the statistics of the firing rates can be neglected, we proved that the probability distributions of the bifurcation points tend to the Gumbel law, and that the statistical properties of large-size multi-population networks can be studied through a powerful dimensional reduction.

For the sake of clarity, we implemented our semi-analytical results for arbitrary-size networks with arbitrary connectivity matrix in the Supplemental Python script “Statistics.py”. The script also performs numerical calculations of the probability distributions of the bifurcation points, of the occurrence probability of the stationary states and of the mean multistability diagram, through which we validated our semi-analytical results. To conclude, in the Supplemental Python script “Permanent.py”, we implemented a comparison between our analytical formula of the permanent for statistically homogeneous multi-population networks, and the Balasubramanian-Bax-Franklin-Glynn algorithm. This comparison proved the higher performance of our formula in the specific case of multi-population networks, provided each external stimulus is shared by a relatively small number of populations.

### 4.1. Progress with Respect to Previous Work on Bifurcation Analysis

In the study of neural circuits, bifurcation theory has been applied mostly to networks composed of graded-output units with analog (rather than discrete) firing rates, see e.g., [53,54,55,56]. On the other hand, bifurcation theory of non-smooth dynamical systems of finite size, including those with discontinuous functions like the discrete network that we studied in this paper, has recently received increased attention in the literature. However, the theory has been developed mostly for continuous-time models [57,58,59,60,61] and for piecewise-smooth continuous maps [62], while discontinuous maps have received much less attention, see e.g., [63]. In [28] we tackled this problem for finite-size networks composed of binary neurons with discontinuous activation function that evolve in discrete-time steps, and we introduced a brute-force algorithm that performs a semi-analytical bifurcation analysis of the model with respect to the external stimuli. Specifically, in [28] we focused on the study of bifurcations in the case of single network realizations. In the present paper we extended those results to networks with quenched disorder, and we introduced methods for performing the bifurcation analysis of the model over network realizations. While in [28] we studied the bifurcations of both the stationary and oscillatory solutions of the network equations, here we focused specifically on the bifurcations of the stationary states, while the study of neural oscillations is discussed in Section 4.3.

Our work is closely related to the study of spin glasses in the zero-temperature limit, since a single realization of our network model has deterministic dynamics. In spin glasses, the physical observables are averaged over the randomness of the couplings in the large-size limit, by means of mathematical techniques such as the *replica trick* and the *cavity method* [10,11,64]. In our work, we followed a different approach, based on extreme value theory and order statistics. This allowed us to reduce the mathematical derivation of the averages and, more generally, of the probability distributions of the stationary states of arbitrary-size networks, to the calculation of 1D definite integrals on the real axis.

To our knowledge, bifurcations of neural networks with quenched disorder were investigated only for fully connected network models with normally distributed weights and graded activation function [16,17,18,19,20]. These studies focused on the thermodynamic limit of the models, preventing us from making progress in the comprehension of the dynamics of small networks, such as microcolumns in the primate cortex [23] or the nervous system of some invertebrates [22]. The neural activity of small networks containing only tens or hundreds of neurons may show unexpected complexity [28]. For this reason, the study of small networks typically requires more advanced mathematical techniques, because the powerful statistical methods used to study large networks do not apply to small ones. Contrary to previous research, in this paper we first focused on the study of networks of arbitrary size, including small ones. Moreover, unlike previous work, we considered networks with an arbitrary synaptic connectivity matrix, which is not necessarily fully connected or normally distributed. In particular, our work advances the tools available for understanding small-size neural circuits, by providing a complete (generally semi-analytical) description of the stationary behavior of Equations (1) and (3). Then, for completeness, and similarly to [16,17,18,19,20], we studied the large-size limit of multi-population networks composed of statistically homogeneous populations. Unlike previous work, which focused on networks composed of graded-output neurons, our binary-rate assumption allowed us to derive asymptotic analytical formulas for the statistics of the stationary states and of the corresponding bifurcation points, advancing our comprehension of neural networks at macroscopic spatial scales.

### 4.2. Limitations of Our Approach

A first limitation of the algorithms that we introduced in the Supplemental file “Statistics.py” is represented by the network size. Note that, during the derivation of our semi-analytical formulas in Section 2.2, we did not make any assumption about the number of neurons in the network. As a consequence, our results are exact for networks of arbitrary size. However, the number of possible firing-rate states in a binary network grows exponentially with the number of neurons, therefore in practice our algorithms can be applied only to small-size networks. The maximum network size that can be studied through our approach depends on the computational power available.

To study the asymptotic statistical properties of large networks, in this paper we focused on the special case of statistically homogeneous networks with arbitrary sparseness and distinct external stimuli to each neural population. The bifurcation points of these networks obey the Fisher-Tippett-Gnedenko theorem, in that they correspond to the extreme values of some conditionally independent and identically distributed random variables. While the extreme value statistics of a finite number of (independent and) non-identically distributed random variables are known (see [36]), a straightforward generalization of the Fisher-Tippett-Gnedenko theorem to statistically heterogeneous variables in the large-size limit is not available [65]. For this reason, a second limitation of our approach is represented by the study of the asymptotic properties of neural networks whose external stimuli are shared by two or more populations. In these networks, the extreme value statistics must be calculated for a set of non-identically distributed random variables, see our discussion at the end of Section 2.4. Therefore the complete characterization of these networks still represents an open problem.

As in [16,17,18,19,20], a third limitation of our work is represented by the assumption of statistical independence of the synaptic connections. The calculation of order statistics for dependent random variables represents another open problem in the literature, which prevents the extension of our results to neural networks with correlated synaptic connections. In computational neuroscience, the dynamical and statistical properties of this special class of neural networks are still poorly understood. A notable exception is represented by [66], which provides a theoretical study of a graded-rate network with correlated normally distributed weights in the thermodynamic limit.

### 4.3. Directions for Future Work on the Statistical Mechanics of Networks with Quenched Disorder

We expect our approach for studying the stationary-state statistics of networks of binary neurons can be extended to more biologically realistic network models. The simplest extensions of the binary model are represented by the study of neuronal models with more complex step functions, that allow the presence of one quiescent state and several firing modes (e.g., networks of ternary neurons, see [67]), and the study of networks of neurons with piecewise-linear activation functions [68]. Because of the piecewise linearity of these models, we expect analytical solutions of their stationary-state statistics can be derived through a generalization of the approach we described in this paper.

However, stationary states represent only a subset of the dynamic repertoire of a network model. Another important dynamical regime in networks with random connections is represented by neural oscillations, which have been previously studied, for example, in [19,69], for fully connected graded-rate neurons in the thermodynamic limit, and for leaky integrate-and-fire neurons, respectively. In future work, we will investigate the possibility to extend our results to the study of neural oscillations in binary network models. Please note that according to [28], the bifurcation points at which an existing neural oscillation disappears, or the formation of a new oscillation is observed, correspond to the minima of minima or to the maxima of maxima of sets of random variables. Provided the arguments of the functions $\min\left(\cdot \right)$ and $\max\left(\cdot \right)$ are conditionally independent given the firing rate ν, it follows that, in principle, the statistics of neural oscillations could be studied (semi-)analytically by applying extreme value theory twice.

### 4.4. Possible Implications of This Work for Circuit Neuroscience

The mathematical techniques developed here may provide useful tools to better understand what are the specific functional advantages of the diversity and heterogeneity of synaptic connections and what it may be their role in stabilizing networks or preventing dysfunctions of brain networks. Being able to better understand mathematically at a deep level how such heterogeneities shape the circuit’s dynamics at different spatial scales, including scales that involve finite-size neural networks, could be thus useful to understand the origin and consequences of aberrant circuit functions.

While much more work is needed to steer results in this direction, our results suggest the presence of strong qualitative and quantitative differences between the behavior or dynamics of the networks with quenched synaptic variability presented here, and networks with similar size and individual neuron models but with homogeneous synaptic weights, that we studied in [28]. The dynamical properties of the networks with quenched disorder change considerably across realizations, as a consequence of their small size. In particular, the variability of the synaptic weights allows the network to explore a larger set of operational modes. For this reason, we typically observe a reorganization of the (mean) bifurcation diagram (in the degree of fragmentation of the diagram in the stimuli space, and in the maximum multistability degree of the stationary solutions), as well as a wider set of accessible stationary states across the network realizations, both for fixed external stimuli and regardless of the stimuli. A systematic study of the enrichment of network dynamics offered by quenched variations in the synaptic strength structures in network models, coupled with more systematic measures of variability of synaptic strengths in healthy or dysfunctional biological neural circuits, could help understanding the possible functional benefits of heterogeneities in neural circuits.

## Figures and Tables

**Table 1:** **An example of network parameters**. This table contains the values of the parameters of the small-size network that we study in Section 3 (see Equation (25) and Figure 1). The symbol × in the matrices C and R means that the statistics of the stationary states and of the bifurcation points are not affected by those parameters, since the corresponding synaptic connections are absent ($P_{i,j}=0$).

	$P=\left[\begin{array}{cccc} 0 & 0.5 & 1 & 0.6\\ 0.4 & 0.5 & 0.1 & 1\\ 0.5 & 0.7 & 0.3 & 0.8\\ 0 & 1 & 0.9 & 0 \end{array}\right],$		$\theta =\left[\begin{array}{c} 0\\ 1\\ 1\\ 2 \end{array}\right]$	
	$C=\left[\begin{array}{cccc} \times & 4 & -3 & -10\\ 6 & 5 & -2 & -4\\ 3 & 4 & -6 & -7\\ \times & 2 & -5 & \times \end{array}\right],$		$R=\left[\begin{array}{cccc} \times & 4 & 2 & 3\\ 5 & 3 & 2 & 3\\ 3 & 4 & 5 & 6\\ \times & 2 & 4 & \times \end{array}\right]$	

**Table 2:** Set of parameters used for generating Figure 5.

Panel A			
N=10,12,⋯,22	X=3	$X_{0}=3$	$Y_{0}=8$
	Y=2	$X_{1}=5$	$Y_{1}=N-8$
		$X_{2}=N-8$	
**Panel B**			
N=16	X=1,2,4,8,16	$X_{\lambda }=\frac{N}{X}$	$Y_{0}=7$
	Y=2	$\forall \lambda \in \left\{0,\cdots ,X-1\right\}$	$Y_{1}=9$

**Table 3:** Set of parameters used for generating Figure 6.

$N_{E}=640$	$\gamma_{E,1}=240$	$\vartheta_{E}=3$	$\mu_{EE}=11$	$\sigma_{EE}=0.8$	$P_{EE}=0.7$
$N_{I}=160$	$\gamma_{I,1}=80$	$\vartheta_{I}=0$	$\mu_{EI}=-8$	$\sigma_{EI}=0.6$	$P_{EI}=0.9$
			$\mu_{IE}=5$	$\sigma_{IE}=0.7$	$P_{IE}=1.0$
			$\mu_{II}=-10$	$\sigma_{II}=0.9$	$P_{II}=0.8$

## Supplementary Materials

The following are available online at https://www.mdpi.com/1099-4300/21/7/630/s1, **File “Statistics.py”:** this Python script calculates semi-analytically the cumulative distribution function of the bifurcation points, the mean multistability diagram, and the occurrence probability of the stationary states (for fixed stimuli and regardless of them) for a network with quenched disorder, according to the methods described in Section 2.2. Moreover, the script compares these results with the numerical evaluation of the corresponding quantities, obtained through the Monte Carlo approach described in Section 2.5. **File “Permanent.py”:** this Python script calculates the permanent of block matrices with homogeneous blocks by means of Equation (20), and compares the computational time of this formula with that of the Balasubramanian-Bax-Franklin-Glynn algorithm.
